# Transcriptomic insights into the roles of the transcription factors Clr1, Clr2 and Clr4 in lignocellulose degradation of the thermophilic fungal platform *Thermothelomyces thermophilus*

**DOI:** 10.3389/fbioe.2023.1279146

**Published:** 2023-10-06

**Authors:** Benedikt Siebecker, Tabea Schütze, Sebastian Spohner, Stefan Haefner, Vera Meyer

**Affiliations:** ^1^ Chair of Applied and Molecular Microbiology, Institute of Biotechnology, Technische Universität Berlin, Berlin, Germany; ^2^ BASF SE, Ludwigshafen, Germany

**Keywords:** *Thermothelomyces thermophilus*, *Myceliophthora thermophila*, cellulases, lignocellulolytic enzymes, transcription factors, transcriptomics, gene regulation, Clr

## Abstract

**Introduction:** *Thermothelomyces thermophilus*, formerly known as *Myceliophthora thermophila*, is used in industry to produce lignocellulolytic enzymes and heterologous proteins. However, the transcriptional network driving the expression of these proteins remains elusive. As a first step to systematically uncover this network, we investigated growth, protein secretion, and transcriptomic fingerprints of strains deficient in the cellulolytic transcriptional regulators Clr1, Clr2, and Clr4, respectively.

**Methods:** The genes encoding Clr1, Clr2, and Clr4 were individually deleted using split marker or the CRISPR/Cas12a technology and the resulting strains as well as the parental strain were cultivated in bioreactors under chemostat conditions using glucose as the carbon source. During steady state conditions, cellulose was added instead of glucose to study the genetic and cellular responses in all four strains to the shift in carbon source availability.

**Results:** Notably, the *clr1* and *clr2* deletion strains were unable to continue to grow on cellulose, demonstrating a key role of both regulators in cellulose catabolism. Their transcriptomic fingerprints uncovered not only a lack of cellulase gene expression but also reduced expression of genes predicted to encode hemicellulases, pectinases, and esterases. In contrast, the growth of the *clr4* deletion strain was very similar compared to the parental strain. However, a much stronger expression of cellulases, hemicellulases, pectinases, and esterases was observed.

**Discussion:** The data gained in this study suggest that both transcriptional regulators Clr1 and Clr2 activate the expression of genes predicted to encode cellulases as well as hemicellulases, pectinases, and esterases. They further suggest that Clr1 controls the basal expression of cellulases and initiates the main lignocellulolytic response to cellulose via induction of *clr2* expression. In contrast, Clr4 seems to act as a repressor of the lignocellulolytic response presumably via controlling *clr2* expression. Comparative transcriptomics in all four strains revealed potentially new regulators in carbohydrate catabolism and lignocellulolytic enzyme expression that define a candidate gene list for future analyses.

## Introduction

Plant biomass, which consists of cellulose (38%–50%), hemicellulose (23%–32%), lignin (12%–25%), and pectin (usually very low percentages), is the most abundant carbon source on Earth and so far the only viable alternative to fossil resources for the production of biofuels and platform chemicals (Cherubini, 2010; Ponnusamy et al., 2018). However, high costs for the pre-treatment and the enzymatic hydrolysis steps, especially high costs for the isolation and purification of the required enzymes, limit commercialization of biochemical lignocellulosic biorefineries (Wertz and Bédué, 2013; Konwar et al., 2018). Filamentous fungi naturally secrete a huge variety of enzymes required for plant biomass degradation, called “CAZYs (Carbohydrate-Active enZYmes)” due to their heterotrophic lifestyle (Lange, 2017; Meyer et al., 2020). Therefore, research on fungal platform strains aims to identify and optimize highly efficient natural enzyme producer strains and to understand CAZY expression in order to tailor and boost the expression of lignocellulolytic enzymes in these hosts.

One of the fungal platform strains of high interest is *Thermothelomyces thermophilus* (formerly known as *Myceliophthora thermophila*), a highly efficient natural secretor of cellulases and hemicellulases. In industry, hypersecreting *T. thermophilus* strains were developed that produce 100–120 g/L of cellulases (Visser et al., 2011; Huuskonen, 2020). The fact that this fungus is a thermophilic fungus is of further interest for lignocellulosic degradation processes, since the secreted enzymes are thermostable (up to 85°C–90 °C) and allow for high process temperatures, which in turn reduce viscosity and thus increase the solubility of lignocellulosic biomass (Viikari et al., 2007; Blumer-Schuette et al., 2014; Berezina et al., 2017; Meyer et al., 2020). Besides cellulase hypersecreting strains, strains with low cellulase expression were also successfully developed in industry with the aim to reduce the natural secretion and therefore allowing for a higher yield, purity, and activity of specific homologous or heterologous proteins (Visser et al., 2011; Haefner et al., 2017b; Haefner et al., 2017a).

It is generally thought that the expression of fungal lignocellulolytic enzymes is mainly regulated at the transcriptional level. Two of the most important activators that are also conserved amongst many filamentous fungi are Clr1 and Clr2 (according to the gene nomenclature in *N. crassa* and *Trichoderma reesei*) or ClrA and ClrB, respectively (according to the gene nomenclature in *Aspergillus* and *Penicillium* species) (Benocci et al., 2017). The regulator Clr1/A occupies a central position and directly regulates genes that are necessary for the hydrolysis of cellulose as well as for the import of soluble degradation products in many filamentous fungi. In *Neurospora crassa* and *Aspergillus nidulans*, Clr1/A is involved in cellulose sensing, as the presence of cellulose or its degradation products (e.g., cellobiose) activate Clr1/A (Coradetti et al., 2012; Coradetti et al., 2013; Craig et al., 2015). Clr2/B regulates genes that are essential for cellulose degradation in *A. nidulans*, *N. crassa*, *Aspergillus niger*, *Aspergillus oryzae* and *Penicillium oxalicum* (Coradetti et al., 2013; Ogawa et al., 2013; Yao et al., 2015; Raulo et al., 2016). To date, *N. crassa* is the sole fungus in which it has been shown that *Clr2* expression is controlled via Clr1, and that both jointly regulate the expression of other transcription factors important for plant biomass degradation such as Col26, Xlr1, and Vib1 (Craig et al., 2015)*.* More recently, Clr4 was identified as another regulator in the Clr family, which was found to activate (hemi)cellulase expression and the expression of other regulators such as Clr1, Clr2 and Xyr1 in *N. crassa* and *T. thermophilus* (Liu et al., 2019).

In the present study, we therefore aimed to lay the groundwork for a systematic understanding of the role of Clr1, Clr2, and Clr4 in the thermophilic platform strain *T. thermophilus*. All studies in literature on lignocellulolytic enzyme expression in *T. thermophilus* rely on data obtained from shake flask experiments, experiments which do not reflect industrial cultivation conditions and are more prone to variation due to less control of variables such as oxygen and pH. We therefore established in this work chemostat cultivation conditions for *T. thermophilus* which allowed us to study growth and physiology of this fungus under non-inducing (glucose as carbon source) and inducing (cellulose as carbon source) conditions in a highly reproducible manner. We furthermore investigated the physiological, transcriptomic and secretion responses of *T. thermophilus* under chemostat conditions using engineered strains carrying deletions for the genes *clr1*, *clr2* or *clr4*.

## Materials and methods

### Strains, media, and growth conditions

To obtain conidia, all *T. thermophilus* strains used in this study (Table 1) were grown at 37 °C on complete medium (CM) (Arentshorst et al., 2012) for 3 days.

**TABLE 1:** Strains used in this study.

Strain	Relevant genotype	References
MJK20.3	Δ*ku80*	Kwon et al. (2019)
BS6.4	Δ*ku80*, Δ*clr2*	This study
BS7.8	Δ*ku80*, Δ*clr1*	This study
JK2.8	Δ*ku80*, Δ*clr4*	This study

For the growth assay, minimal medium (MM) (Arentshorst et al., 2012) with bromophenolblue as a pH indicator was used. For mono- and disaccharides a final concentration of 25 mM and for polysaccharides a final concentration of 1% in the medium were used. Mono- and disaccharides were sterile filtrated and added to the medium after autoclaving. Galacturonic acid, polygalacturonic acid, and pectin were added prior to autoclaving and the pH was adjusted to ∼ pH 5.5. Cellulose and xylan were added to the medium before autoclaving without adjusting the pH value. 1,000 spores (in 10 μL) of each strain were spotted on MM agar and incubated at 37 °C for 4 days.

All bacterial plasmids were propagated in *Escherichia coli* TOP10 (Invitrogen) using 100 μg/mL ampicillin or 50 μg/mL kanamycin for selection.

### Bioreactor cultivation and analysis

For bioreactor cultivation New Brunswick BioFlo310 bioreactors (Eppendorf) were used. Prior to this, a protocol for a stable bioreactor cultivation had to be established. During this process, several hurdles had to be overcome (data not shown). In brief, too high a concentration of calcium chloride (2.7 mM CaCl_2_x2H_2_O) and too low a temperature (37 °C) were causing (independently from each other) a sporulation in batch phase when less than 10% of the supplied glucose were consumed. After adjusting the calcium chloride concentration to 0.27 mM CaCl_2_x2H_2_O and the cultivation temperature to 45°C, a stable batch cultivation without premature sporulation was possible and the chemostat cultivations were started. For this purpose, 1*10^9^ spores/L were inoculated in bioreactor medium containing 76 mM (NH_4_)_2_SO_4_, 2 mM MgSO_4_x7H_2_O, 12 mM KH_2_PO_4_, 7.5 mM KCl, 0.27 mM CaCl_2_x2H_2_O, 0.025 mM biotin, 55.5 mM glucose and trace elements (134 µM EDTA disodium salt dihydrate, 70 µM ZnSO_4_x7H_2_O, 162 µM H_3_BO_3_, 23 µM MnSO_4_xH_2_O, 16.4 µM FeSO_4_x7H_2_O, 6.5 µM CoCl_2_x6H_2_O, 5.8 µM CuSO_4_x5H_2_O, 5.7 µM Na_2_MoO_4_x2H_2_O; pH adjusted to 6 using 1 M NaOH). CaCl_2_x2H_2_O, glucose and trace elements were autoclaved separately and added to the medium after autoclaving. Biotin was sterile filtrated and added to the medium after autoclaving. Prior to autoclaving pH was set to 6.7 using 10 M NaOH. Prior to inoculation, the temperature was set to 45°C, pH value to 6.7 (if necessary), stirring to 100 rpm, and aeration to 0.01 slpm (after reaching 100% DOT). pH regulation was achieved via 25% ammonia solution and 20% phosphoric acid. After inoculation, a time-based profile for aeration and stirring was started. Aeration and stirring were constantly increased within 10 h to 1 slpm and 750 rpm, respectively. At the end of the exponential phase when glucose concentration was close to 0–1 g/L (∼13 g base addition) chemostat cultivation was started by addition of medium at a dilution rate of 0.1 1/h and maintaining culture broth weight at 5 kg. To prevent foaming, PPG 2000 was added with a rate of 20 mg/h during chemostat cultivation. In steady state, the carbon source was changed to cellulose (autoclaved separately) by removing 500 g culture broth and subsequently adding 450 g bioreactor medium (without glucose) and 50 g microcrystalline cellulose resulting in 5 kg total weight of the culture broth. Cultivation was continued as a batch cultivation. Offgas values (O_2_ consumed, CO_2_ produced), temperature, pH, dissolved oxygen (DO), weight, and base addition were monitored during cultivation. Samples for biomass, protein and glucose determination, RNA extraction, and microscopy were taken before and after (0.5 h, 1 h, 2 h, 4 h) the addition of the medium containing cellulose. Every sample taken was split into different aliquots according to the planned analysis. For microscopy, a small amount of the culture broth was transferred to a reaction tube. The biomass sample was gained by filtrating the culture broth sample via vacuum filtration, collecting mycelium on a filter to obtain dry biomass weight. The supernatant of that sample was used for protein and glucose determination. Samples for RNA isolation were taken as described for the biomass sample, but the filter carrying the mycelium was immediately frozen in liquid nitrogen.

### Molecular biology methods

Most molecular techniques were performed according to standard procedures described in (Green and Sambrook, 2012) if not mentioned separately. Transformation of *T. thermophilus* and isolation of genomic DNA were performed as described in (Arentshorst et al., 2012) with exceptions described in (Kwon et al., 2019). Primers and plasmids used in this study are summarized in Supplementary Material S11 and Supplementary Material S12. All Plasmids were obtained by BASF SE except plasmid pBS1.13 for the deletion of *clr4*. This plasmid was generated via circular polymerase extension cloning (CPEC) (Quan and Tian, 2011) using the primers listed in Supplementary Material S11 to generate the different fragments (see Supplementary Material S1) used for CPEC. The structure and content of each deletion cassette was similar for all plasmids except the respective 5′and 3′flanks for homologous recombination.

The progenitor strains of all other mentioned strains were MJK20.2 (Kwon et al., 2019) or MJK20.3 (both Δ*ku80*). MJK20.3 was generated by sub cultivating and re-analyzing MJK20.2 since wildtype contamination was detected in MJK20.2 (due to sensitivity, only detectable with diagnostic PCR not via Southern analysis, data not shown).

The regulator deletion mutants were generated by deleting the respective genes (*clr1*: MYCTH_2298863, *clr2*: MYCTH_38704, and *clr4*: MYCTH_2296492) in MJK20.2 or MJK20.3. Strain BS5.14 (Δ*ku80*, *clr2*:DR-P*AngpdA*–An*amdS*-TAn*amdS*-DR) was generated via transforming 3 µg of each PCR-amplified split marker fragment (see Supplementary Material S11 and Supplementary Material S12 for plasmids and primers used) containing an *amdS* marker and approximately 1 kb flanks each for homologous recombination. The 3′split marker fragment contained a second 5′flank for *amdS* marker removal. Strains BS7.8 (Δ*ku80*, Δ*clr1*) and JK1.8 (Δ*ku80*, *clr4*:DR-P*AngpdA*–An*amdS*-TAn*amdS*-DR) were generated via an RNP based genome editing approach using Cas12a (Kwon et al., 2019) (see Supplementary Material S11 and Supplementary Material S12 for plasmid and primers used). For BS7.8 3 µg of each split marker fragment and for JK1.8 3 µg of the whole plasmid carrying the deletion cassette was used instead of two split marker fragments.

After transformation, the resulting strains BS5.14 and JK1.8 were sub-cultivated on fluoroacetamide (FAA) medium plates according to (Arentshorst et al., 2012) to obtain marker free strains resulting in strains BS6.4 (Δ*ku80*, Δ*clr2*) and JK2.8 (Δ*ku80*, Δ*clr4*). For BS7.8 counterselection on FAA medium was not necessary because sub cultivation already allowed for *amdS* marker removal.

Strains were analyzed via diagnostic PCR and Southern blot analysis to verify the correct integration, the absence of wildtype contamination, and removal of the marker gene. The results of the Southern blot analysis of the marker recycled strains are shown in Supplementary Material S1.

### Biochemical methods

Protein concentration was determined via Bio-Rad Protein Assay Kit II (Bio-Rad) according to the manufacturer’s instructions. Absorbance was measured at 595 nm and bovine serum albumin was used as a reference.

SDS PAGEs were performed using a 12.5% resolving gel and a 5% stacking gel. Samples were prepared via using a defined volume (20 μL) or a defined amount (2 μg) of protein. This was achieved by either freeze drying the respective volume or the respective amount (based on the results of the protein concentration determination) of the sample and adding 10 μL of H_2_O MQ.

Glucose concentration was determined using the Glucose Fluid GOD-PAP Kit (Mti Diagnostics) according to the manufacturer’s instructions with 10 µL sample and 100 µL reagent volume. Absorbance was measured at 505 nm.

### RNA isolation, purification, sequencing, and analysis

RNA was isolated with the Trizol reagent (Invitrogen) using frozen ground mycelium according to the manufacturer’s instructions.

Samples were purified using the innuPREP RNA Mini Kit 2.0 (Analytik Jena) according to the manufacturer’s instructions. Possible remaining DNA was removed via DNA-free™ DNA Removal Kit (Invitrogen) according to the manufacturer’s instructions. After purification and DNAse treatment, samples were ready for RNA sequencing.

RNA was sequenced at Microsynth AG (Balgach) for strains MJK20.3 and BS6.4 and at GenomeScan (Leiden) for strains BS7.8 and JK2.8 using an Illumina platform with 150 bp reads paired end, polyA enrichment, and >5 million reads per sample. A quality check of the RNA samples prior to sequencing was included according to the guidelines of the company. Prior to RNA sequencing at GenomeScan (Leiden), no purification and DNAse treatment was performed.

Obtained read data were first quality controlled via FastQC (Andrews, 2010) and if necessary, trimmed with BBTools (Bushnell, 2014). STAR (Dobin et al., 2013) was used for mapping the reads to the *T. thermophilus* ATCC 42464 genome (assembly ASM22609v1, downloaded from NCBI (https://www.ncbi.nlm.nih.gov/)). Data normalization and differential gene expression analysis was performed with DEseq2 (Love et al., 2014). Differential gene expression was evaluated with Wald test and Benjamini and Hochberg False Discovery Rate (FDR) with a threshold of 0.05 (Benjamini and Hochberg, 1995). Enrichment analyses were performed with DAVID (Huang et al., 2009b; 2009a) using standard settings and a *p*-value cutoff of 0.05. Gene annotations were obtained combining information from DAVID (see above), NCBI (see above), JGI (https://jgi.doe.gov/), the CAZY database (http://www.cazy.org/) and publications (Berka et al., 2011; Karnaouri et al., 2014). Venn analysis was done with the help of http://bioinformatics.psb.ugent.be/webtools/Venn/. Packages used for analysis, creating plots, and diagrams in R via RStudio (https://rstudio.com/) are “complexheatmap”, “circlize”, “devtools”, “rafalib”, “rsamtools”, “BiocParallel”, “DESeq2”, “GenomicFeatures”, “GenomicAlignments”, “ggplot2”, “pheatmap”, “RColorBrewer”, and “gplot”. RNA Seq. raw and processed data have been deposited at the GEO database (https://www.ncbi.nlm.nih.gov/geo/) under the accession number GSE183387. Supplementary Material S13 includes the raw and normalized read counts.

For quantitative polymerase chain reaction (qPCR), purified and DNAse treated RNA was transcribed into cDNA (RevertAid H Minus First Strand cDNA Synthesis Kit; Thermo Fischer Scientific). The qPCR reaction was performed using the Biozym Blue S´Green qPCR Kit (Biozym) according to the manufacturer’s instructions. No template controls (NTC) and no reverse transcriptase controls (NRT) were included. The primers used for the reaction are listed in Supplementary Material S11. The results are shown in Supplementary Material S4.

## Results

### Generation of *clr1*, *clr2,* and *clr4* deletion strains

The general deletion strategy in *T. thermophilus* was based on a split marker approach using the removable *amdS* as selection marker (Kelly and Hynes, 1985; Nielsen et al., 2006) as well as the CRISPR/Cas12a technology established in our lab (Kwon et al., 2019) (for details see Supplementary Material S1 and Materials and Methods). Strain MJK20.3, which is deleted for the *ku80* gene that encodes a component of the non-homologous end joining machinery (Critchlow and Jackson, 1998) and thus allows highly efficient targeted homologous recombination frequencies (Kwon et al., 2019), was used as parental strain. In this strain, the genes encoding the transcription factors Clr1, Clr2, or Clr4 were individually deleted using the *amdS* marker. The genotypes were verified after marker removal via diagnostic PCR (data not shown) and Southern analysis (Supplementary Material S1). The resulting strains were named BS7.8 (*Δclr1*), BS6.4 (*Δclr2*), and JK2.8 (*Δclr4*) (Table 1).

**FIGURE 1:**
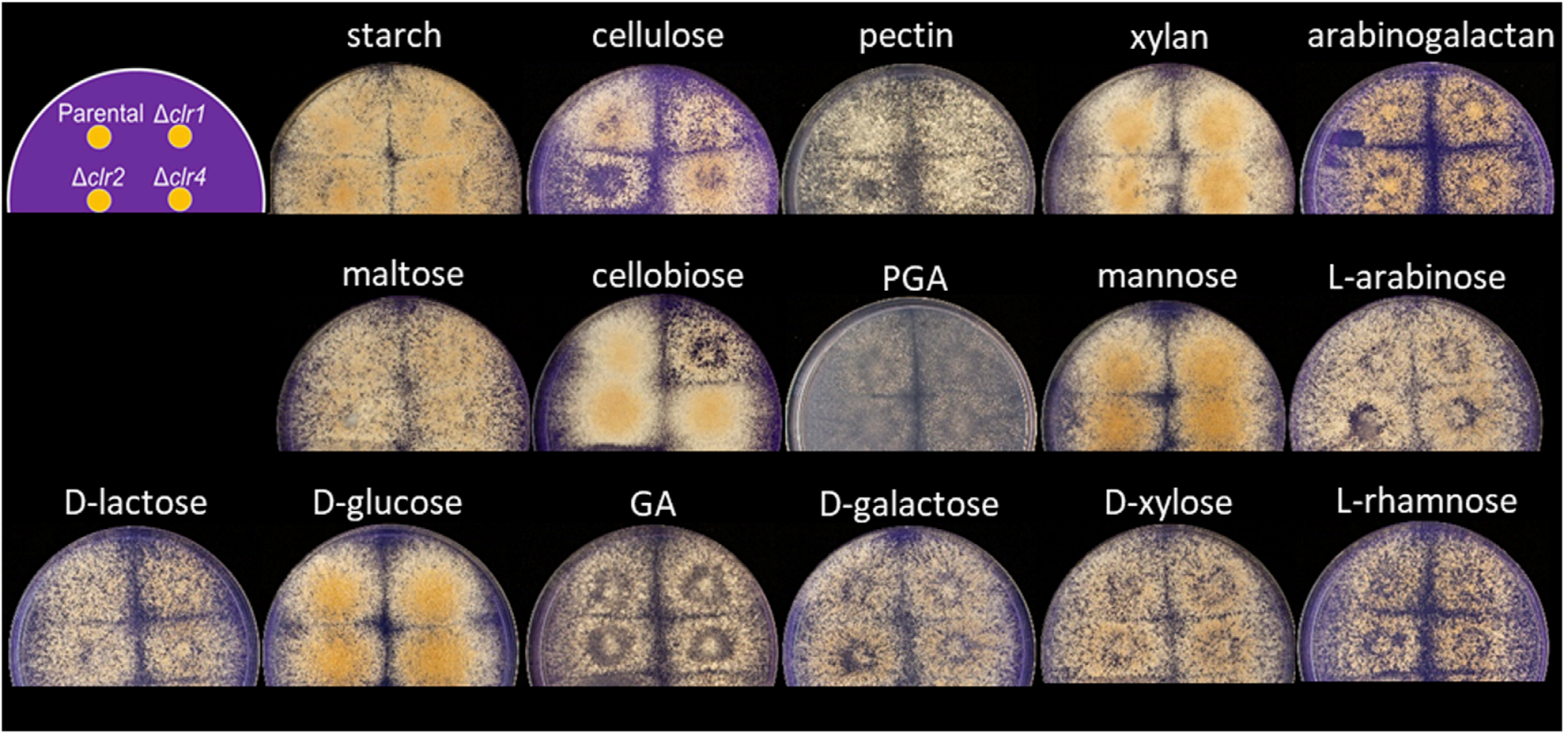
Carbon-source dependent growth of *T. thermophilus* strains deleted for *clr1*, *clr2* or *clr4*, respectively. Minimal medium agar plates were inoculated with 1,000 spores for the strains MJK20.3, BS7.8 (*Δclr1*), BS6.4 (*Δclr2*) or JK2.8 (*Δclr4*) according to the scheme shown in the upper left. Pictures were taken after 4 days of cultivation at 37 °C. GA = galacturonic acid, PGA = polygalacturonic acid.

To test whether the different regulators are important for *T. thermophilus* to feed on monomeric and polymeric carbon sources derived from plant biomass, a growth assay was conducted with the parental and the deletion strains. As depicted in Figure 1, all strains were able to grow on the 16 different carbon sources tested. However, BS6.4 (*Δclr2*) displayed reduced growth on cellulose and pectin and BS7.8 (*Δclr1*) on cellulose and cellobiose, respectively. Notably, *T. thermophilus* prefers glucose, cellobiose, xylan, mannose, starch, and cellulose over the other carbon sources tested.

### Physiology of *T. thermophilus* strains during glucose-limited chemostat cultivations

**FIGURE 2:**
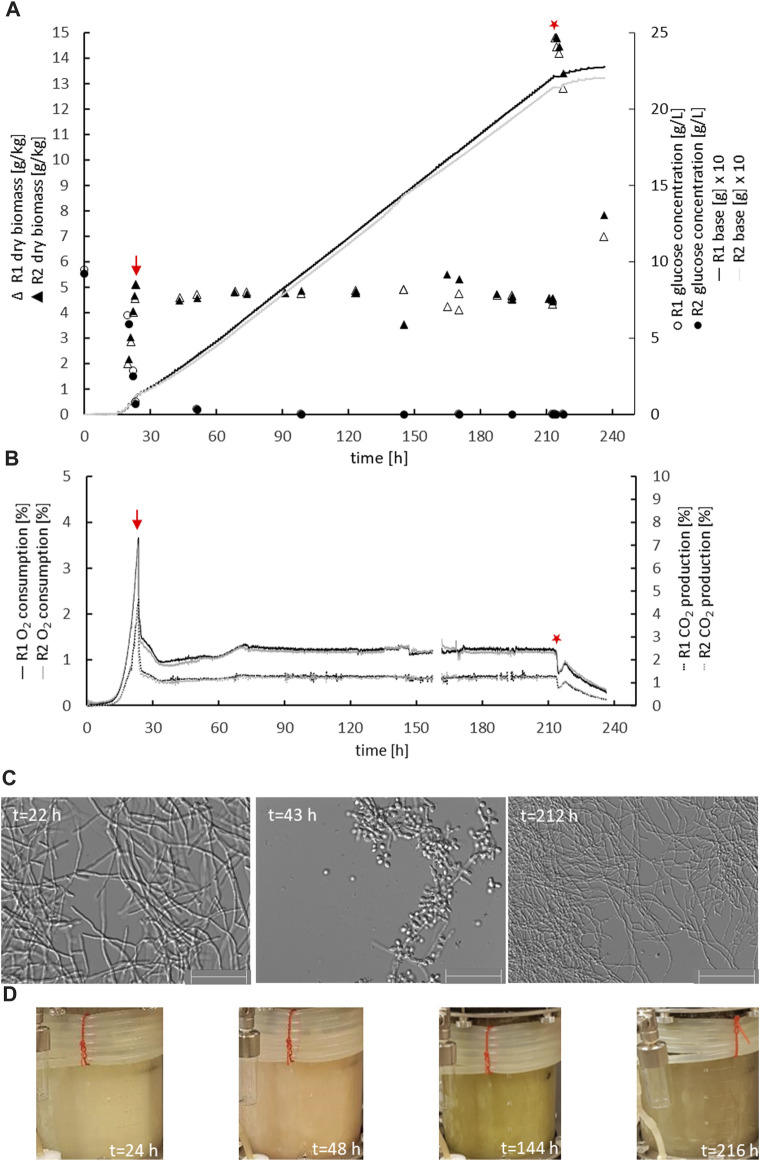
Physiology of the parental strain during chemostat bioreactor cultivation. Biomass accumulation, glucose concentration, base addition **(A)**, oxygen consumption, carbon dioxide production **(B)**, mycelial morphology **(C)**, and colour of the culture broth **(D)** are given for duplicate cultures of strain MJK20.3 (R1, R2). The chemostat cultivation mode was started at the end of the batch phase, indicated with a red arrow. After steady state conditions were reached, 1% cellulose was spiked instead of glucose (red star) and chemostat cultivation was switched to a batch cultivation mode. Note that the addition of cellulose caused an immediate increase in culture dry weight of 10 g/kg and a short-term drop in off gas values. After this, base addition automatically continued, off gas values raised again and biomass decreased, which demonstrated the ability of the control strain to use cellulose as a carbon source. The colour of the culture broth shifted from white-greyish (exponential growth) over pinkish (sporulation after starting chemostat initiation) to brown-greyish (during steady state condition). Scale bar = 50 µm.

**FIGURE 3:**
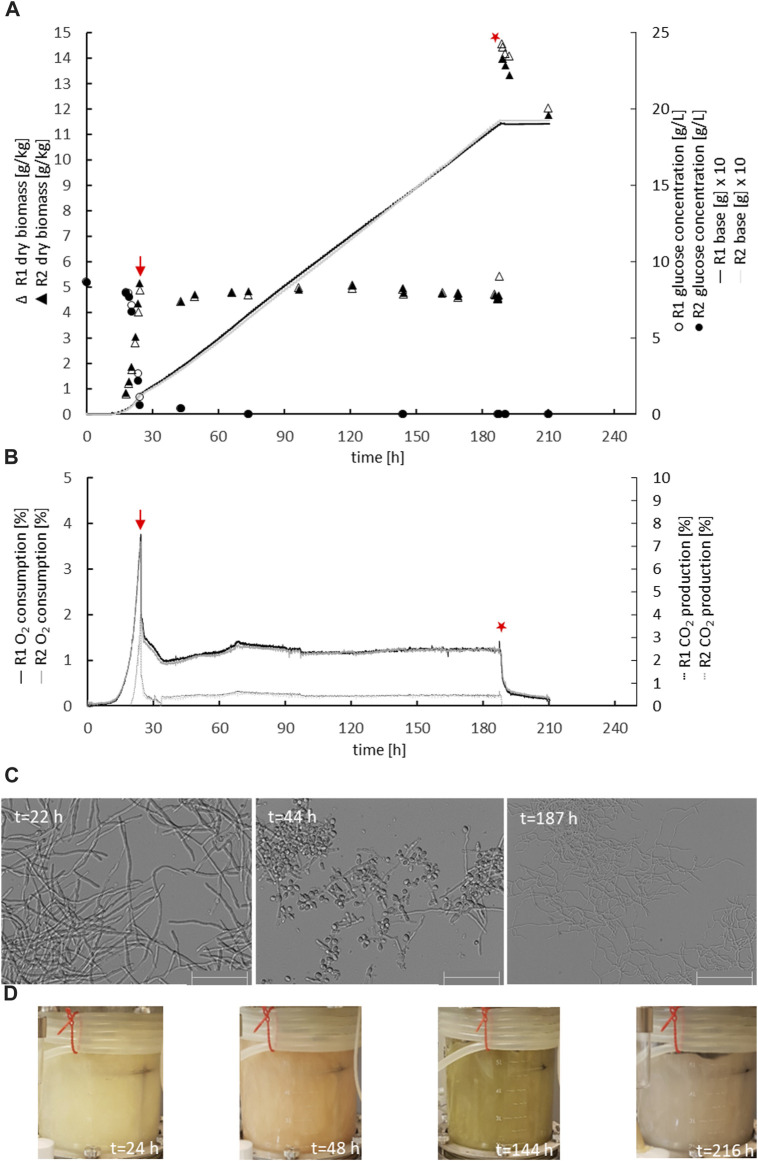
Physiology of the *clr2* deletion strain during chemostat bioreactor cultivation. Strain BS6.4 was cultivated and analysed as described in Figure legend 2. Biomass accumulation, glucose concentration, base addition **(A)**, oxygen consumption, carbon dioxide production **(B)**, mycelial morphology **(C)**, and colour of the culture broth **(D)** are given for duplicate cultures of strain BS6.4 (R1, R2). Scale bar = 50 µm.

The parental reference strain MJK20.3 was used to establish a glucose-limited chemostat cultivation protocol. During the late exponential growth phase, when the biomass reached 5 g_DCW_/kg, the cultivation process was switched to the chemostat mode with a dilution rate D = 0.1 h^−1^. Importantly, this switch to glucose-limited feeding provoked a brief sporulation period for about 10 h, after which *T. thermophilus* slowly resumed filamentous growth which was stable until the end of the chemostat runs (Figure 2 A-C; Table 2). Steady state conditions, where biomass, base addition, and off-gas values (CO_2_ produced, O_2_ consumed) were constant for at least 5 residence times, were achieved after about 12–17 residence times. Therefore, strain MJK20.3 and the engineered deletion strains for *clr1*, *clr2*, and *clr4*, respectively, were run in duplicate glucose-limited chemostat cultures for approximately 210 h, after which cellulose was added to the bioreactor medium instead of glucose and the cultivation program switched to a batch mode (for details see Materials and Methods). The reference strain as well as strain JK2.8 (Δ*clr4*) were able to feed on the newly added cellulose and thus started a new exponential growth phase (Figure 2 A-C and Supplementary Material S2; Figures 1A–C), whereas the strains deleted for *clr1* and *clr2*, respectively, were unable to consume cellulose and thus stopped growing (Figure 3 A-C and Supplementary Material S2; Figures 2A–C). Color development of the fermentation broth was comparable between all cultivated strains (Figures 2, 3 D). The maximum growth rates for all strains were very similar, as well as their biomass in steady state (Table 2). Protein secretion as well as specific production rate of extracellular protein in steady state differed especially between JK2.8 (Δ*clr4*) and the other strains (Table 2). Hyphal diameters in exponential state as well as in steady state were very similar between the four strains (Table 2). Nevertheless, notably smaller hyphal diameters could be detected during steady state conditions when compared to the respective diameters in exponential state (Table 2).

**TABLE 2:** Physiological data for the strains MJK20.3, BS7.8 (*Δclr1*), BS6.4 (*Δclr2*) and JK2.8 (*Δclr4*) during bioreactor cultivation. Standard deviations (±) are given for mean values of duplicate independent results. µ_exponential state_: maximum growth rate in exponential state; C_biomass_: biomass concentration in steady state as dry cell weight (DCW); C_protein_: protein concentration in steady state; q_protein_: specific production rate of extracellular protein in steady state. Hyphal diameters in exponential state and steady state were measured from at least 50 individual hyphae.

	Parental	Δ*clr1*		Δ*clr2*		Δ*clr4*
**µ**_**exponential state**_ **(h** ^**-1**^ **)**	0.27 ± 0.01	0.29 ± 0.01		0.29 ± 0.01		0.27 ± 0.00
**C**_**biomass**_ (**g**_**DCW**_ **/kg)**	4.53 ± 0.03	4.58 ± 0.18		4.55 ± 0.03		4.61 ± 0.12
**C**_**protein**_ **(µg/µL)**	89.92 ± 5.89	73.65 ± 3.61		65.08 ± 8.49		58.23 ± 2.51
**q**_**protein**_ (**C**_**protein**_ **/C**_**biomass**_)	19.84 ± 1.30	16.07 ± 0.79		14.30 ± 1.86		12.64 ± 0.54
**hyphal diameter**_**exponential state**_ **(µm)**	2.66 ± 0.33	2.89 ± 0.33		2.53 ± 0.25		2.94 ± 0.32
**hyphal diameter**_**steady state**_ **(µm)**	1.51 ± 0.21	1.54 ± 0.22		1.24 ± 0.17		1.63 ± 0.21

**FIGURE 4:**
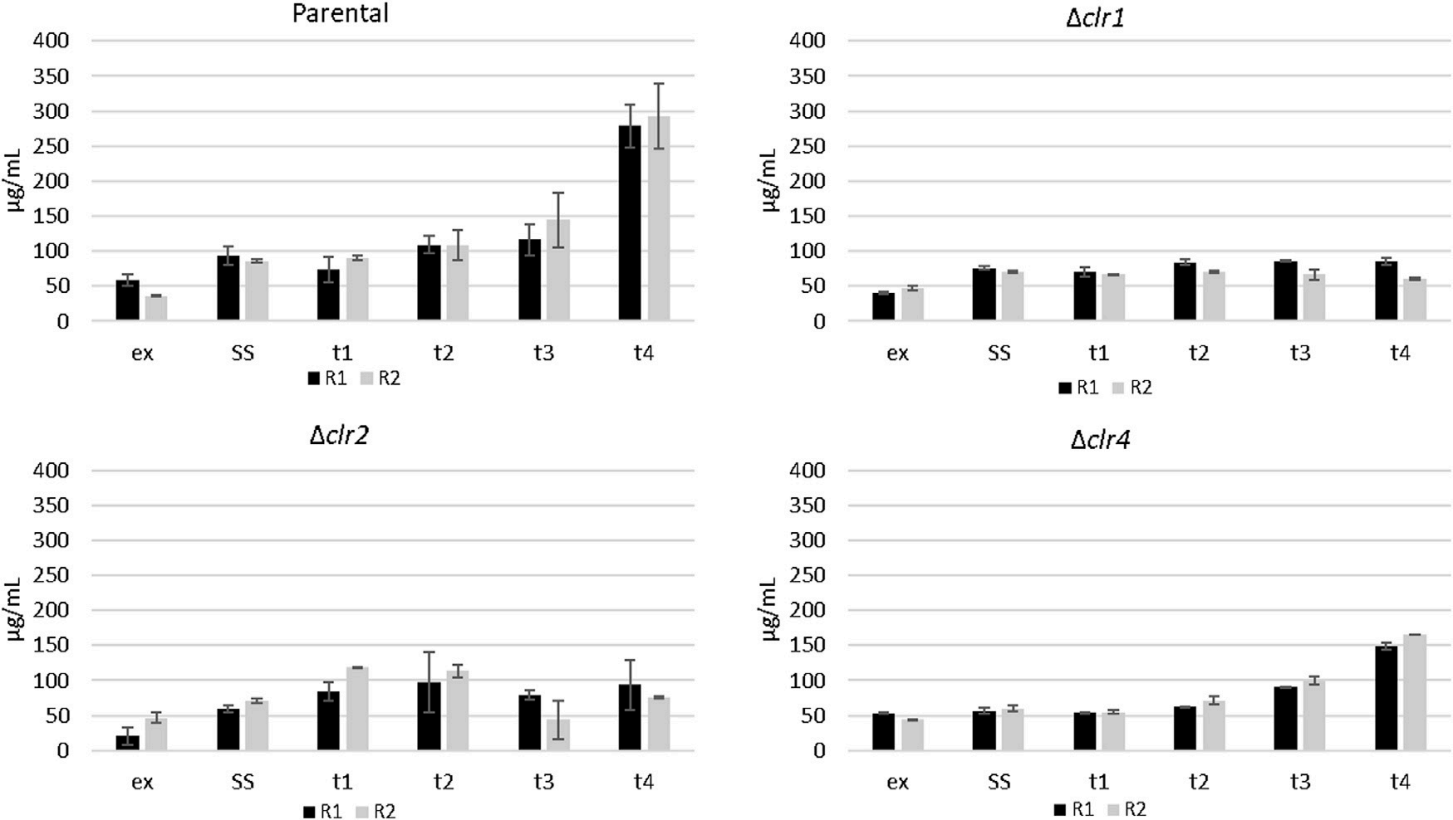
Protein secretion in *T. thermophilus* strains deleted for *clr1*, *clr2* or *clr4* in response to cellulose. The amount of protein in the culture supernatants of the samples taken in exponential state (ex), steady state (SS), as well as 0.5 h (t1), 1 h (t2), 2 h (t3), and 4 h after spiking cellulose (t4) for the two replicates (R1, R2) was determined via Bradford assay. The error bar represents the standard deviation of the mean value, deriving from three technical replicates for each biological duplicate sample.

To investigate a potential impact of cellulose feeding on protein secretion in the 4 *T. thermophilus* strains, proteins were isolated from the culture supernatants of all strains during steady state conditions as well as 30 min, 1 h, 2 h, and 4 h after the cellulose spike. An increase of approximately 200 mg/L (5 fold vs steady state) and 100 mg/L (3 fold vs steady state) secreted protein was detected for the parental and the *Δclr4* strain, respectively (Figure 4), suggesting that the transcription factor Clr4 is important to fully induce protein secretion in response to ambient cellulose. Notably, an altered protein secretion profile became visible especially 4 h after the cellulose spike in the parental and the *Δclr4* strain (Supplementary Material S3). The *clr1* and *clr2* deletion strains did not show any change in their secreted protein titres when confronted with cellulose (Figure 4), demonstrating that both strains - in contrast to the strain deleted for *clr4* - were not able to adapt to the new polymeric carbon source and suggesting that the transcription factors Clr1 and Clr2 are key for the adaptational response to cellulose.

### Global transcriptomic responses of *T. thermophilus* to cellulose adaptation

RNA-sequencing (RNA-Seq.) analysis using samples extracted from duplicate chemostat cultures corresponding to steady state conditions and 30 min, 1 h, 2h, and 4 h, respectively, after the cellulose spike was performed for all three deletion strains and the parental strain. All 40 samples were normalized to allow for direct comparison and used for differential gene expression analyses using moderated t-statistics with a false discovery rate (FDR) < 0.05 (see Methods). Principal component analysis (PCA) demonstrated that steady state samples from all strains clustered together. As expected, cellulose samples from the parental and *Δclr4* strain clustered together, as did cellulose samples for the *Δ clr1* and *Δ clr2* strains (Supplementary Material S4). Quantitative real-time PCR performed for four exemplarily selected genes of our interest confirmed the RNA sequencing data (Supplementary Material S4). The complete list of differentially expressed genes of all sample comparisons including log_2_fold change and statistical significance is given in Supplementary Material S5. Several thousand genes out of the 9,292 predicted *T. thermophilus* genes were identified as differentially expressed upon the shift to cellulose relative to the respective steady state condition (Table 3). Notably, the number of differentially expressed genes were higher in all deletion strains when compared to the parental strain MJK20.3 with highest numbers in the *Δclr1* strain followed by the *Δclr2* strain (Table 3). Venn diagrams uncovered that 345/146 (parental), 1,271/1,238 (*Δclr1*), 983/1,053 (*Δclr2*), and 465/225 (*Δclr4*) genes were up-/downregulated across all points in time after the cellulose spike (Supplementary Material S6). Based on these results, the gene sets were deemed important to study in a more detailed manner. We thus performed GO term enrichment analysis with these gene sets as described earlier (Paege et al., 2016) and in the Methods section (Supplementary Material S7). We note, however, that only ∼30–45% of all *T. thermophilus* genes do have a GO term annotation depending on the GO term category (https://david.ncifcrf.gov/(Huang et al., 2009b; 2009a)). Consequently, fold enrichment values can be very high despite the low number of genes that belong to a respective category. As summarized in Supplementary Material S7, processes being enriched in the upregulated gene sets of the parental and the *Δclr4* strains upon cellulose adaptation belonged to “cellulose catabolic process”, “xylan catabolic process”, “cellulase activity”, “xylanase activity”, and “pectate lyase activity”, whereas processes being enriched in the downregulated gene sets included “carbon metabolic process” and “transferase activity”. The transcriptional response of the deletion strains *Δclr1* and *Δclr2*, which were not able to continue growth after the carbon shift from glucose to cellulose, showed accordingly enriched GO terms connected to cell death in the upregulated gene sets (“mitophagy”, “late nucleophagy”, and “autophagy”) and enriched GO terms connected to growth in the downregulated gene sets (“respiration”, “replication”, “transcription”, “translation”, “biosynthesis”).

**TABLE 3:** Differentially expressed genes in *T. thermophilus* strains in response to the cellulose spike. Number of differentially expressed genes related to the respective steady state condition with a padj. ≤ 0.05, 0.5 h (t1), 1 h (t2), 2 h (t3), and 4 h (t4) after the cellulose spike compared to the respective steady state condition.

Strain	Sample	Upregulated genes	Downregulated genes
MJK20.3 **(parental)**	t1	1,419	1,376
	t2	1797	1721
	t3	1,297	1,308
	t4	604	455
BS7.8 **(*Δclr1*)**	t1	2,311	2063
	t2	2,467	2,174
	t3	2,436	2,251
	t4	2,285	2,238
BS6.4 **(*Δclr2*)**	t1	1981	1936
	t2	2,398	2,232
	t3	2,228	2057
	t4	2012	1939
JK2.8 **(*Δclr4*)**	t1	1734	1,673
	t2	2,102	2052
	t3	1,517	1,535
	t4	733	601

### Transcriptomic response of predicted carbohydrate-hydrolysing enzymes

**FIGURE 5:**
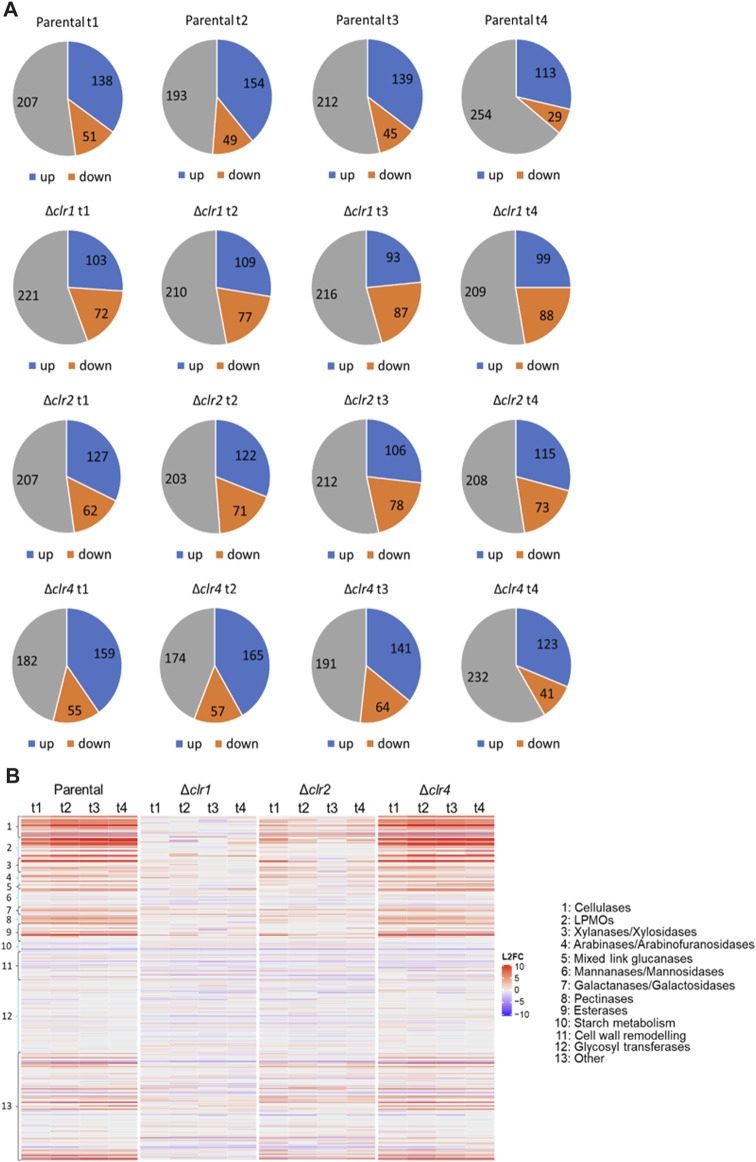
Number of differentially expressed genes predicted to encode CAZYs **(A)** Numbers of up- (blue) and downregulated (orange) genes as well as genes with no differential expression (grey) at 0.5 h (t1), 1 h (t2), 2 h (t3), and 4 h (t4) after the cellulose spike compared to the respective steady state condition **(B)** Heatmap showing the respective log2 fold change values of these genes (L2FC) belonging to different CAZY classes. Negative values (blue) represent downregulated and positive values (red) upregulated genes.

In order to specifically understand transcriptomic adaptations in *T. thermophilus’* carbohydrate metabolism to the shift from glucose to cellulose, annotations of CAZYs predicted in the genome of *T. thermophilus* were retrieved from the databases JGI (https://jgi.doe.gov/), NCBI (https://www.ncbi.nlm.nih.gov/) and CAZY (http://www.cazy.org/) as well as from published literature (Berka et al., 2011; Karnaouri et al., 2014). In total, 396 predicted CAZY genes were retrieved, which were divided into different classes and types according to their function and the type of organic carbon they degrade. Genes that could not be assigned to a specific CAZY class or type were grouped into the class “other” (Supplementary Material S8). As depicted in Figure 5, CAZY expression in the parental and *Δclr4* strains is almost identical with a slightly stronger differential expression for some upregulated genes especially in the cellulase, hemicellulase, pectinase, and esterase categories in the *Δclr4* strain. Genes that are predicted to function in starch metabolism showed strongest downregulation. In contrast, nearly all predicted cellulase, hemicellulase, pectinase, esterase and “other” encoding genes that were strongly upregulated in the parental and *Δclr4* strains, were not or only moderately upregulated in *the Δclr2* strain, and not differentially expressed in the *Δclr1* strain (Figure 5B).

**FIGURE 6:**
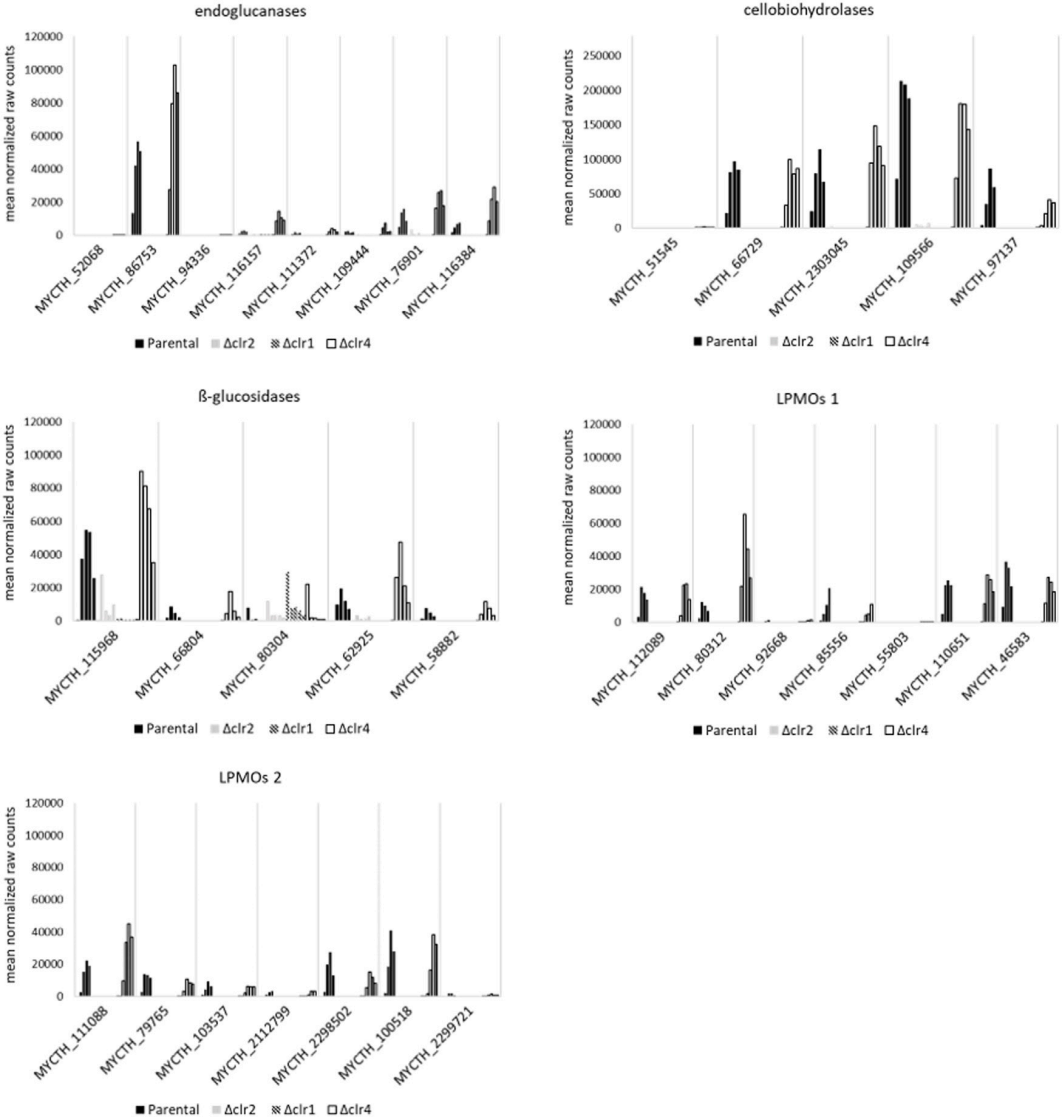
Expression of predicted cellulase genes in response to cellulose. Shown are the mean values of the normalized raw counts of the two replicates at steady state, 0.5 h, 1 h, 2 h, and 4 h after spiking with cellulose (left to right bar) for cellulases that are differentially expressed at all points in time after the cellulose spike in *T. thermophilus* strains deleted for *clr1* (patterned), *clr2* (grey), or *clr4* (black, empty) in comparison to the parental strain (black, filled).

We individually analysed this set of 113 differentially expressed genes including 17 cellulases to identify candidate genes that are supposedly under strongest control of the three transcription factors Clr1, Clr2, or Clr4. Of interest are the predicted endoglucanases MYCTH_86753, MYCTH_76901, MYCTH_116384, and MYCTH_116157, because they were upregulated at highest level in the parental and *Δclr4* strains but not in the *Δclr1* and *Δclr2* strains (Figure 6 and Supplementary Material S9). Among the predicted cellobiohydrolases, the four highest expressed upregulated genes in the parental and *Δclr4* strains but not in the *Δclr1* and *Δclr2* strains were MYCTH_109566, MYCTH_97137, MYCTH_66729, and MYCTH_2303045 (Figure 6). Notably, none of these genes showed expression in the *Δclr1* and *Δclr2* strains, except for MYCTH_109566, which showed some residual expression in the *Δclr2* strain. The highest expressed and upregulated predicted ß-glucosidase genes were MYCTH_115968, MYCTH_62925, and MYCTH_66804. No expression of these genes was observed in the *Δclr1* and *Δclr2* strains with exceptions for MYCTH_115968 (the strongest expressed ß-glucosidase in the parental strain) and MYCTH_62925 in *Δclr2*, where a residual expression can be observed (Figure 6). Three genes predicted to encode lytic polysaccharide monooxygenases (LPMOs) were very highly expressed and upregulated in the parental and *Δclr4* strains (MYCTH_80312, MYCTH_111088, MYCTH_112089, Figure 6). Notably, MYCTH_80312 and MYCTH_111088 showed about 2-4-fold higher expression in the *Δclr4* strain, when compared to the parental strain, whereas five other predicted LPMO genes (MYCTH_85556, MYCTH_46583, MYCTH_2298502, MYCTH_100518, and MYCTH_79765) showed slightly higher expression in the parental strain when compared to the *Δclr4* strain. None of these LPMOs showed expression neither in the *Δclr1* nor in the *Δclr2* strain (Figure 6). Such a trend for cellulase-encoding genes was also observed for the categories hemicellulases, pectinases, and esterases (Supplementary Material S8 and Supplementary Material S9), i.e., many genes showed higher expression levels in the *Δclr4* strain when compared to the parental strain but very low or no expression in the *Δclr1* and *Δclr2* strains.

### Transcriptomic response of predicted transcription factors

**FIGURE 7:**
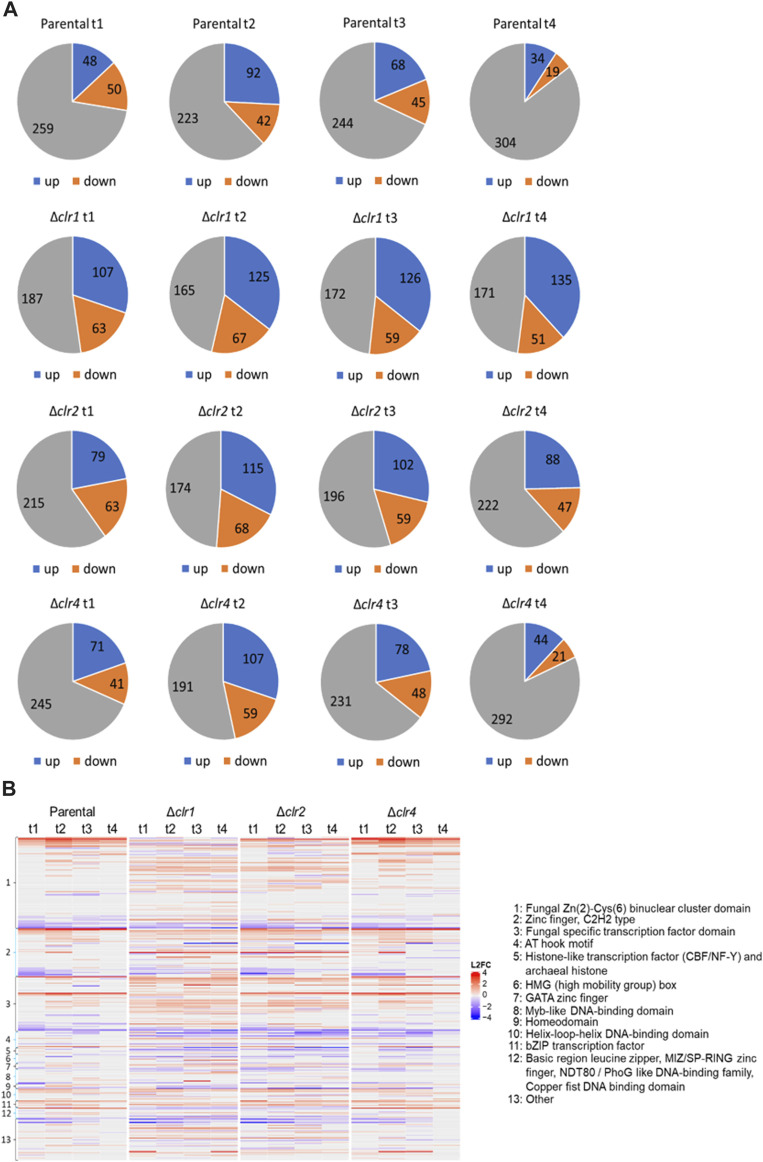
Number of differentially expressed genes predicted to encode transcription factors. **(A)** Numbers of up- (blue) and downregulated (orange) genes as well as genes with no differential expression (grey) at 0.5 h (t1), 1 h (t2), 2 h (t3), and 4 h (t4) after the cellulose spike compared to the respective steady state condition **(B)** Heatmap showing the respective log2 fold change values of these genes (L2FC) belonging to different transcription factor classes. Negative values (blue) represent downregulated genes and positive values (red) upregulated genes.

Annotations of predicted transcription factor genes of *T. thermophilus* were retrieved from the databases JGI (https://jgi.doe.gov/), NCBI (https://www.ncbi.nlm.nih.gov/) and published literature. In total, 357 genes were retrieved to potentially encode transcription factors, which were grouped into different classes according to their DNA binding domain (Supplementary Material S10). Since transcription factors can have multiple DNA binding domains, single transcription factors can be found in several classes. Classes with less than two members were grouped together in the category “other”. Transcription factors that were up- or downregulated across all time points after the cellulose spike, were for us of highest interest as these presumably control the lignocellulolytic response to cellulose. Up to 30% of the predicted transcription factors showed differential expression in the parental strain after the shift to cellulose (∼130), which increased to about 50% of the predicted transcription factors in the *Δclr1* and *Δclr2* strains (∼190), again suggesting that both Clr1 and Clr2 are of fundamental regulatory importance for *T. thermophilus* to feed on cellulose (Figure 7A). A less dramatic effect was seen in the *Δclr4* strain, which is congruent with a nearly identical heatmap when compared to the parental strain but considerably different when compared to both *Δclr1* and *Δclr2* strains, respectively (Figure 7B). Notably, strongest differential expression were observed for genes predicted to encode fungal-specific Zn (2)-Cys (6) binuclear cluster domain transcription factors (Figure 7B).

**FIGURE 8:**
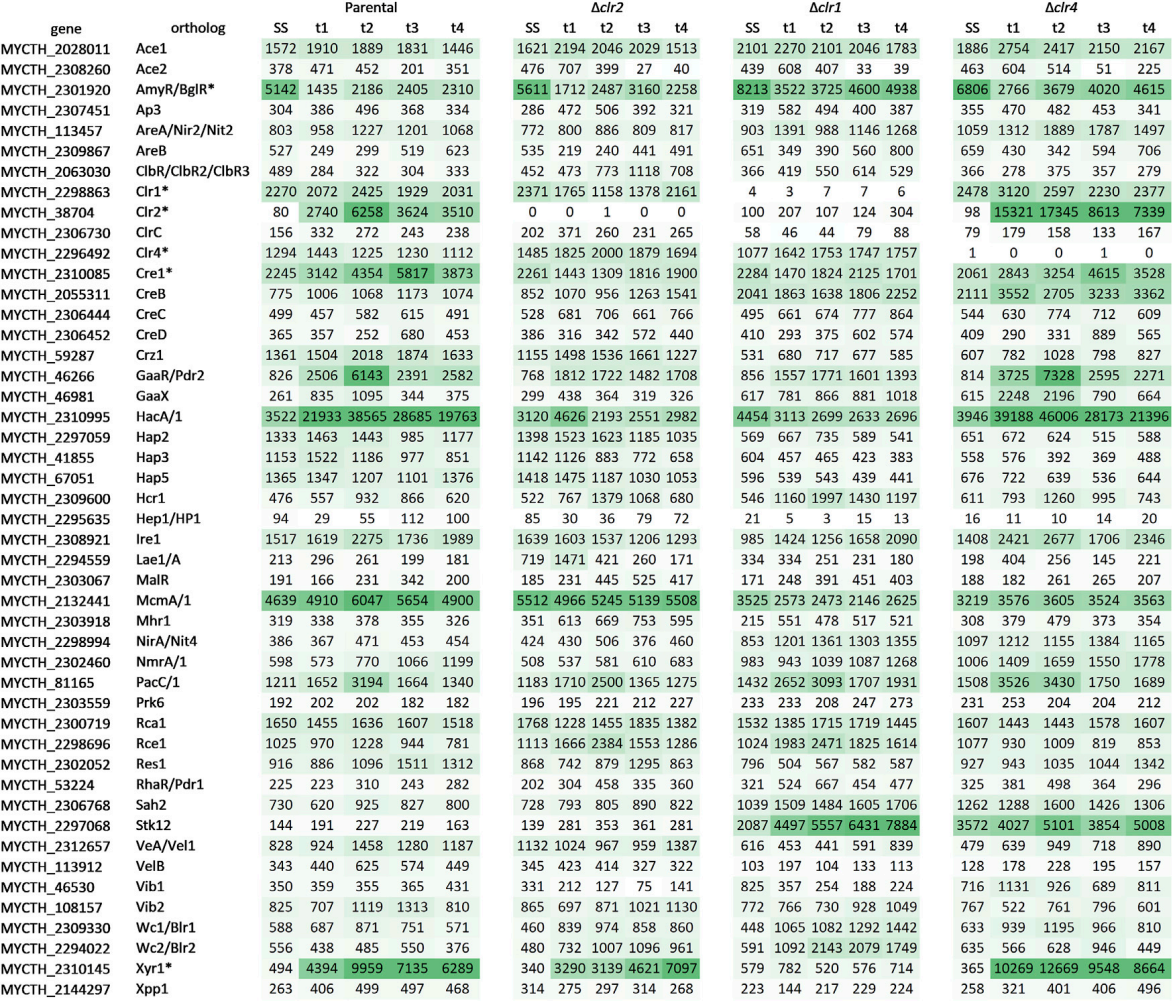
Qualitative heatmap with expression profiles of orthologous genes predicted to encode fungal transcription factors involved in carbon degradation. Shown are the mean values of the normalized raw counts of the two replicates at steady state (SS) as well as 0.5 h (t1), 1 h (t2), 2 h (t3), and 4 h (t4) after the cellulose spike compared to the respective steady state condition. The green colour scales with the expression level of the gene (the darker the higher). A dash separates possible orthologs of this regulator if more than one was found. An asterisk marks regulators that have already been investigated in *T. thermophilus*. The original data including the respective log2 fold change values can be found in Supplementary Material S10.

Although the function of the majority of transcription factors is unknown in *T. thermophilus*, we specifically scrutinized the expression pattern of 47 orthologs of known filamentous fungal transcription factors involved in carbon metabolism and growth control (Figure 8). Lack of any expression of *clr1*, *clr2*, or *clr4* in the respective deletion strains confirmed their successful deletions in strains BS7.8, BS6.4, and JK2.8, respectively. Highest expression as well as upregulation in the parental and the Δ*clr4* strain were observed for MYCTH_38704 (Clr2), MYCTH_2310085 (Cre1), MYCTH_46266 (GaaR/Pdr2), MYCTH_2310995 (Hac1/A), and MYCTH_2310145 (Xyr1) (Figure 8). From these, all except MYCTH_2310085 (Cre1) were more strongly expressed in the Δ*clr4* strain, and conversely more weakly expressed in the *Δclr1* and *Δclr2* strains. A further exception is MYCTH_2310145 (Xyr1), which displayed higher expression after 4 h in the Δ*clr2* strain. Notably, MYCTH_2310145 (Xyr1) showed nearly no expression in the Δ*clr1* strain but was highly expressed in the three other strains during all time points. Transcription factor encoding genes that displayed high expression levels although they were not differentially expressed in all four strains (or only at one point in time after the cellulose spike) included MYCTH_2298863 (Clr1), MYCTH_2296492 (Clr4) and MYCTH_2132441 (McmA/1) (Figure 8). From these, MYCTH_2298863 (Clr1) was stronger and MYCTH_2132441 (McmA/1) more weakly expressed in the *Δclr4* strain when compared to the parental strain. In contrast, MYCTH_2132441 (McmA/1) was more highly expressed in the *Δclr2* strain (t1, t4 only) and much more weakly expressed in the *Δclr1* strain when compared to the parental strain. For another predicted transcription factor, MYCTH_2297068 (Stk12), the opposite trend was observed, i.e., a strong expression in the *Δclr1* and *Δclr4* strains but not in the *Δclr2* and parental strains. The data altogether imply that within the regulatory network i) Clr1 could potentially regulate *clr2* expression, which is implied by the low expression levels of *clr2* in the Δ*clr1* strain, ii) Clr4 might act as a transcriptional brake, which is implied by the much higher expression levels of, e.g., *clr2*, *xyr1*, and *hacA/1* in the Δ*clr4* strain and iii) McmA/1 and Stk12 might play an important regulatory role for cellulase expression.

## Discussion

The experimental approach followed in this study enabled us to obtain and compare physiological and transcriptomic fingerprints for strains deleted for the transcriptional regulators Clr1, Clr2, and Clr4 with their parental *T. thermophilus* strain. The results from mutually confirming data obtained from PCA plot analyses, differential gene expression analyses, GO term enrichment analyses, protein analyses and growth assays indicate an important role of these transcription factors for the expression of lignocellulosic enzymes including cellulases, hemicellulases, pectinases, and esterases.

### The role of Clr1 in *T. thermophilus*

We could confirm a key role of Clr1 for growth on cellulose and cellobiose as described earlier for *N. crassa* (Coradetti et al., 2012) and *T. thermophilus* (Haefner et al., 2017a). Nearly all predicted cellulase, hemicellulase, pectinase, and esterase genes, which became upregulated in the parental strain in response to cellulose, failed to do so in the strain deleted for *clr1*. Nevertheless, growth on glucose was unaffected in the *clr1* deletion strain. Based on the observations in this study, *clr1* seems to be constitutively expressed. As described for *N. crassa* and *A. nidulans*, Clr1 becomes activated via inducers only (e.g., cellulose) (Coradetti et al., 2013) and *clr1* overexpression does not lead to expression of Clr1 target genes in *N. crassa* (Coradetti et al., 2012; Craig et al., 2015). It remains to be shown in future studies whether this is also the case in *T. thermophilus*. Our data further suggest that the transcriptional factors Clr2, Cre1, Xyr1, GaaR/Pdr2, HacA/1, and McmA/1 are under direct or indirect control of Clr1 in *T. thermophilus*. Regarding regulation of Clr2, so far it has only been shown in *N. crassa* that Clr1 controls *clr2* expression (Coradetti et al., 2012; Coradetti et al., 2013). A more detailed discussion regarding the Clr1 dependent regulation of *clr2* follows in the next section of the discussion. The weaker expression of *cre1* compared to the parental strain in *T. thermophilus* is likely caused by the inability of the *clr1* deletion strain to degrade cellulose and thus not releasing glucose which itself acts as an inducer of CreA/1 as shown in *A. nidulans* and *N. crassa* (Orejas et al., 1999; Orejas et al., 2001; Tamayo et al., 2008; Sun and Glass, 2011). Clr1 dependent *xlr1* expression as observed in this study (*clr1* deletion strain) was also described for *N. crassa* (Craig et al., 2015), *T. thermophilus* (Haefner et al., 2017a) and *Aspergilli* (Raulo et al., 2016) and was accompanied by reduced expression of predicted hemicellulase genes known to be under control of Xyr1. It was earlier proposed that Xyr1 is presumably not involved in cellulose degradation in *T. thermophilus* (Dos Santos Gomes et al., 2019). However, in this study we observed a very high expression level of *xyr1* in the parental strain after spiking with cellulose. This fits well to the observed strong expression of predicted hemicellulase and acetyl esterase genes, whose expression could be regulated by Xyr1 as already shown for predicted xylanase genes in *T. thermophilus* (Dos Santos Gomes et al., 2019). Hemicellulase expression in *T. thermophilus* might therefore be coupled with cellulase expression. GaaR/Pdr2 is known to be exclusively involved in pectin degradation and not described so far to be regulated via ClrA/1 in filamentous fungi (Alazi et al., 2016; Niu et al., 2017). As the *T. thermophilus clr1* deletion strain expresses less of the ortholog of GaaR/Pdr2 and also less of predicted pectin lyase genes compared to the parental strain, we suggest that cellulase and pectinase genes are co-regulated by Clr1 similar to hemicellulose gene expression (see above). The importance of HacA/1 for balancing protein secretion during the lignocellulolytic response was already earlier described for filamentous fungi (Huberman et al., 2016) and might also be the case for *T. thermophilus*. A weaker expression of the HacA/1 ortholog is in good agreement with the observed lower secretion in the *clr1* deletion strain, meaning that the unfolded protein response is less needed when compared to the parental strain where high expression of CAZYs and thus a high protein secretion load occurs. McmA/1 is known to positively control cellulase expression presumably via interaction with ClrB*/2* in *A. nidulans*, but has no impact on cellulase production in *Talaromyces cellulolyticus* (Yamakawa et al., 2013; Tani et al., 2014; Fujii et al., 2015). Due to the high constitutive expression of *mcmA/1* and its transcriptional dependency of *clr1* expression, a similar function compared to Clr1, or even a transcriptional regulation of *mcmA/1* via Clr1, might be possible to allow fine tuning of cellulase expression in *T. thermophilus*. Finally, the gene predicted to encode the ortholog of *N. crassa* Stk12 is much more highly expressed in the *clr1* deletion strain when compared to the parental strain. The function of Stk12 could be similar as reported for *N. crassa*, where the deletion of *stk12* resulted in a 7-fold higher cellulase production compared to the wildtype (Lin et al., 2019).

### The role of Clr2 besides Clr1 in *T. thermophilus*

The phenotypic and transcriptomic consequences for *T. thermophilus* when deleted for *clr2* were very similar compared to the *clr1* deletion strain, with the exception that the *clr2* deletion strain is still able to grow on cellobiose. An importance of Clr2 for growth on cellulose was already observed in *N. crassa* (Coradetti et al., 2012). Furthermore, *T. thermophilus* was shown to have a reduced ability to secrete proteins when deleted for *clr2* (Haefner et al., 2017b). This fits to the finding that the DNA-binding domain of Clr2 in *T. thermophilus* was recently shown to be important for the response to cellulose (Zhang et al., 2022). Our transcriptomic data uncovered why cellobiose can still be utilized as a carbon source after the deletion of *clr2*: the predicted ß-glucosidase gene MYCTH_115968, the highest expressed predicted ß-glucosidase gene in the parental strain, is still expressed in the Δ*clr2* strain although at a low level. The importance and high level expression of this ß-glucosidase during cellulose degradation was also shown by previous transcriptomic analysis experiments. Here it was also shown that this ß-glucosidase has the highest expression levels after cellulose induction similar to our observations (Qin et al., 2022). Due to such residual expression of some predicted cellulase, hemicellulase, pectinase, and esterase genes like MYCTH_115968 in the Δ*clr2* strain (which is not the case in the Δ*clr1* strain), we speculate that i) Clr1 is important for a basal expression of these lignocellulolytic enzymes to ensure their expression as scouting enzymes and that ii) the main lignocellulolytic response might become two-step triggered via Clr1-dependent expression of *clr2*. Future studies which will analyse *clr1, clr2* double deletion strains can affectively assess this hypothesis. Another remarkable difference between the Δ*clr1* and Δ*clr2* strains is expression of genes encoding for the orthologs of McmA/1, Stk12, and Xyr1. The genes encoding McmA/1 as well as Stk12 are regulated by Clr1 in *T. thermophilus* but likely not by Clr2 since similar expression levels were observed in the *clr2* deletion strain when compared to the parental strain. Based on these observations and published literature (see previous section) we speculate that Clr1 and McmA/1 could have overlapping functions in *T. thermophilus* and could potentially regulate each other’s gene expression to enable fine tuning of cellulase expression. As, however, the expression trend of *mcmA/1* and *stk12* behaves contrary in the *clr1* deletion strain, they could have opposing functions. These two hypotheses are worth studying further. Finally, expression of *xyr1* in the Δ*clr2* strain is also very different compared to its expression in the Δ*clr1* strain. *Xyr1* expression is absent in the *clr1* deletion strain but detectable in the clr2 deletion strain, implying that Clr1 could be directly or indirectly the main regulator of xyr1 expression.

### The role of Clr4 besides Clr1 and Clr2 in *T. thermophilus*

This study suggests that besides Clr1 and Clr2, Clr4 is also of importance for cellulase expression in *T. thermophilus*. A considerable number of predicted cellulase, hemicellulase, pectinase, and esterase genes that become upregulated in the parental strain upon the shift to cellulose (especially the highest expressed ones) display a much higher expression in the Δ*clr4* deletion strain. This is accompanied by upregulation of genes predicted to encode transcription factors (e.g., *clr2*, *gaaR/pdr2*, *hacA/1*, and *xyr1*). We propose that the higher expression of these regulators and CAZYs in the *clr4* mutant is presumably because of the much higher expression of *clr2*. In agreement, it was recently shown that Clr4 is able to bind to *clr2* promotor sequences in *T. thermophilus* and *N. crassa* (Liu et al., 2019). Interestingly, expression trends of the orthologs of Stk12 and McmA/1 are similar in the *clr1* and *clr4* deletion strains but not when compared to the parental strain. Therefore, it could be conceivable that a direct or indirect interaction between Clr1 and Clr4, besides the possibility of an independent trigger by the single transcription factors, could potentially regulate the expression of *stk12* and *mcmA/1*. Future experiments could unravel whether Clr1 and Clr4 could potentially co-coordinate carbon-catabolite repression and fine-tuning of lignocellulolytic enzyme expression via these regulators in *T. thermophilus*. In addition, further deletion experiments are necessary to clarify whether the orthologs of McmA/1 and Stk12 are inducers or repressors of cellulase expression. Due to the constitutive expression of the *clr4* gene, we furthermore propose that Clr4 could, similar to Clr1, require an inducer to become activated as a transcription factor. Intriguingly, upregulation of genes predicted to encode transcription factors (e.g., *clr2*, *gaaR/pdr2*, *hacA/1*, and *xyr1*) and lignocellulolytic enzymes in the *clr4* deletion strain could either suggest that Clr4 acts as a repressor, i.e., as a transcriptional brake, of this lignocellulolytic network and/or as an activator of other genes encoding hydrolytic enzymes that enter the secretory pathway and block it otherwise for secretion of lignocellulolytic enzymes. Notably, the data obtained for the *Δclr4* deletion strain in this study deviate from data published earlier for *T. thermophilus* and *N. crassa* obtained from shake flask cultures (Liu et al., 2019). There, protein band patterns observed in SDS-PAGE analyses differed considerably from the reference strain used. Also, much less secreted proteins were observed in the deletion strain when cultivated on cellulose, and *clr2* and *xyr1* showed reduced expression. We assume that this is because of the different experimental setup and sampling times used. As the transcriptional response to changing carbon sources is a very fast and dynamic one, usually followed by homeostatic feedback loop mechanisms generally inherent to all biological systems, it will become an important question for future experiments to dissect the time-dependent responses of *T. thermophilus* to cellulose.

## Conclusion

This study uncovered that cellulase gene expression is tightly coupled with hemicellulase, pectinase, and esterase gene expression in *T. thermophilus* when cultivated on cellulose. The transcription factors Clr1 and Clr2 are the main regulators of these CAZYs, and presumably perform this function by co-regulating other transcriptional factors including Xyr1, GaaR/Pdr2, Stk12 and McmA/1 to name but a few. The data suggest that Clr1 ensures basal expression of cellulases irrespective of the presence of cellulose and that *clr2* expression requires Clr1. When *T. thermophilus* becomes confronted with cellulose as main carbon source, Clr1 initiates the main lignocellulolytic response via Clr2. Finally, the results of this study suggest that Clr4 acts as a repressor of cellulase expression presumably via regulation of *clr2* expression.

## Data Availability

The datasets presented in this study can be found in online repositories. The names of the repository/repositories and accession number(s) can be found in the article/Supplementary Material.

## Supplementary material

SUPPLEMENTARY MATERIAL S1 clr1, clr2, and clr4 deletion and marker removal.

SUPPLEMENTARY MATERIAL S2 Bioreactor cultivations (*clr1* and *clr4* deletion strains).

SUPPLEMENTARY MATERIAL S3 Secreted protein profiles of all four strains.

SUPPLEMENTARY MATERIAL S4 PCA and quantitative real-time PCR.

SUPPLEMENTARY MATERIAL S5 Candidate genes important for cellulose adaptation.

SUPPLEMENTARY MATERIAL S6 Venn diagrams.

SUPPLEMENTARY MATERIAL S7 GO term enrichment analysis.

SUPPLEMENTARY MATERIAL S8 List of CAZYs and expression values.

SUPPLEMENTARY MATERIAL S9 Differentially expressed CAZY categories.

SUPPLEMENTARY MATERIAL S10 List of transcription factors and expression values.

SUPPLEMENTARY MATERIAL S11 Oligonucleotides used in this study.

SUPPLEMENTARY MATERIAL S12 Plasmids used in this study.

SUPPLEMENTARY MATERIAL S13 Raw counts, normalized counts, DE values all genes.
