# Supplementary material for: Transcriptomic insights into the roles of the transcription factors Clr1, Clr2 and Clr4 in lignocellulose degradation of the thermophilic fungal platform Thermothelomyces thermophilus
Source: Front Bioeng Biotechnol. 2023 Oct 6;11:1279146. doi: 10.3389/fbioe.2023.1279146 (PMC10588483; doi:10.3389/fbioe.2023.1279146)
Supplement: Supplementary file 1 [file Table1.DOCX]

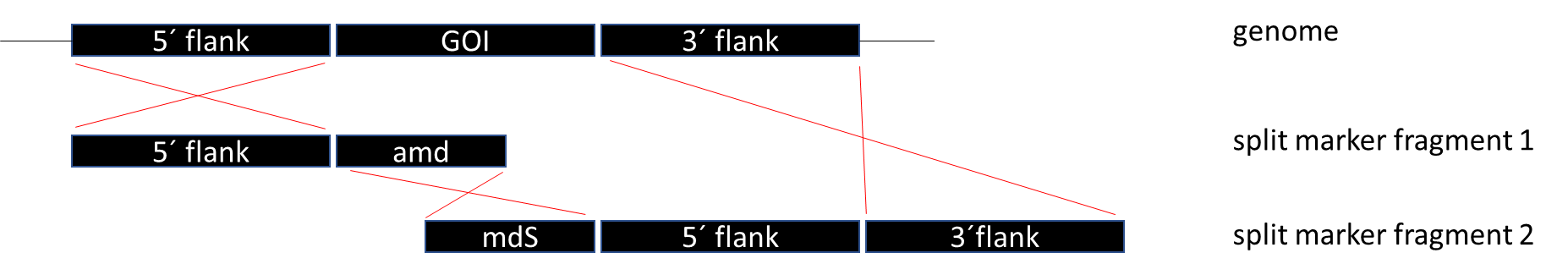

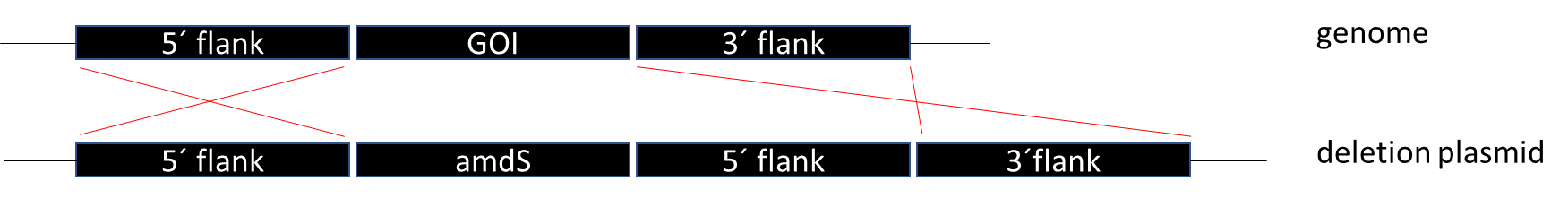

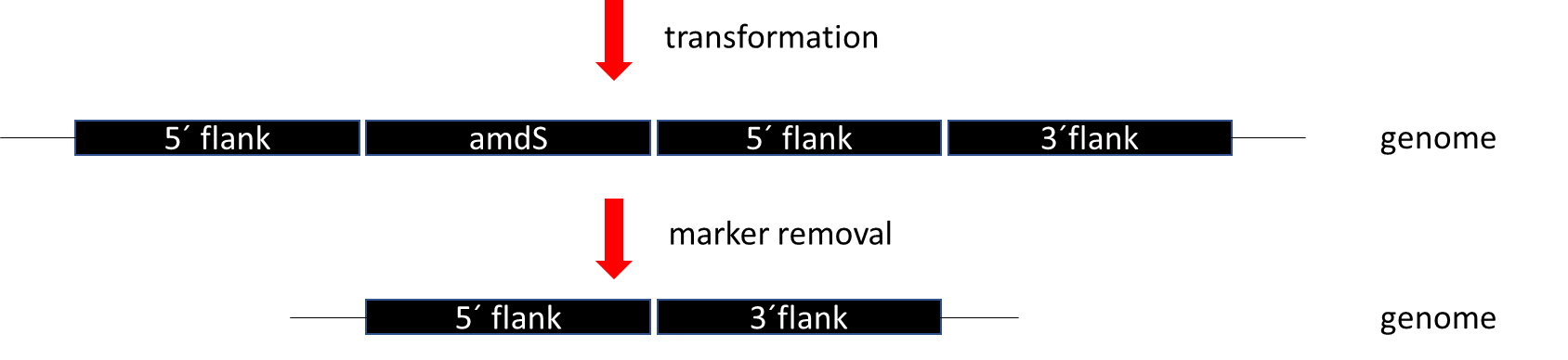


**A**

**B**

**C**

**D**

**S1 Figure 1: Schematic illustration of gene deletion strategies used in this work. Homologous recombination sites are shown as red lines.** (A) Split marker approach. Two split marker fragments were transformed in T. thermophilus to enhance the possibility of correct homologous recombination, resulting in (C). (B) CRISPR/Cas12a approach. The whole plasmid carrying the deletion cassette was transformed in T. thermophilus, where CRISPR/Cas12a (depicted by scissors) was used to cut in the gene of interest (GOI) to enhance the possibility of correct homologous recombination. This approach also resulted in (C). After the gene was successfully deleted (C), the marker (amdS) was removed via FAA plating, resulting in (D).

S1 Figure 2

**
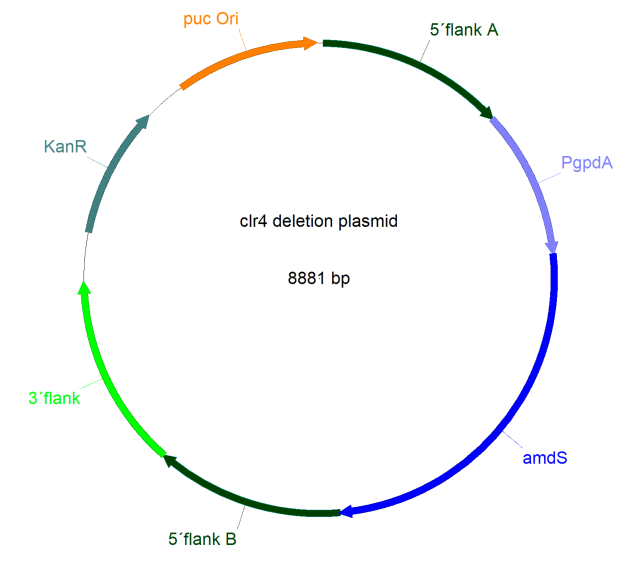
**

**S1 Figure 2: Map of the plasmid used for clr4 deletion.** The plasmid is carrying a 5´ flank (A) and a 3´flank, surrounding the clr4 ORF for homologous integration of the via PgpdA from A. nidulans constitutively expressed amdS selection marker gene (including terminator) and a second 5´flank (B) for marker removal. KanR and puc Ori are required for selection and amplification in E. coli.

| fragment | template used to amplify fragment | primer used to amplify fragment | fused fragments | primer used to fuse fragments |
| --- | --- | --- | --- | --- |
| BB | MT134 | 2132+2089 | BB+5´flank A | 2132+2136 |
| 5´flank A | gDNA | 2128+2081 |  |  |
| P An *gpdA–*An *amdS*-T An *amdS* | pMJK19.7 | 2129+2083 |  |  |
| 5´flank B | gDNA | 2130+2085 | 5´flank B+3´flank | 2130+2139 |
| 3´flank | gDNA | 2131+2087 |  |  |

**S1 Table 1: Templates and primers used to amplify fragments and fused fragments for CPEC.** The fused fragments and the amdS selection marker fragment were subsequently used in CPEC. BB= backbone. An= Aspergillus nidulans.


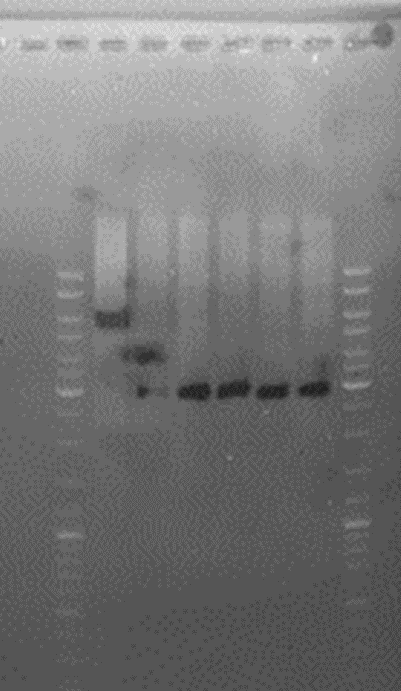

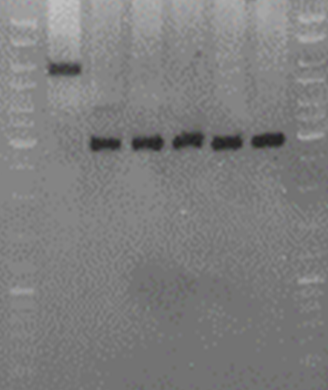


3000 bp

2500 bp

6000 bp

5000 bp

M

M

M

M

MJK20.3

BS7.14

BS7.13

BS7.10

BS7.8

BS7.4

MJK20.3

BS7.14

BS7.13

BS7.10

BS7.8

BS7.4

**A**

**B**

**S1 Figure 3: Deletion of clr1 in MJK20.3.** Shown are the results of the Southern analysis after marker recycling using a 5´probe (A) and a 3´probe (B). Enzymatic digestion was performed using XmnI. A marker (M) was used as a reference for fragment sizes. The expected fragment sizes are shown in S1 Table 2.


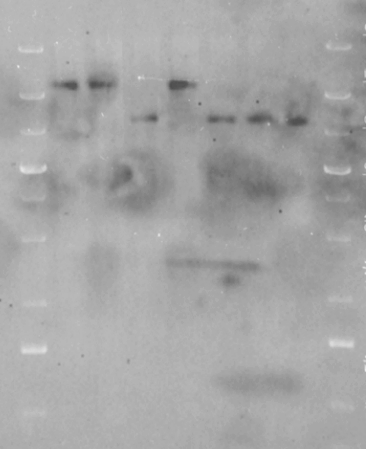

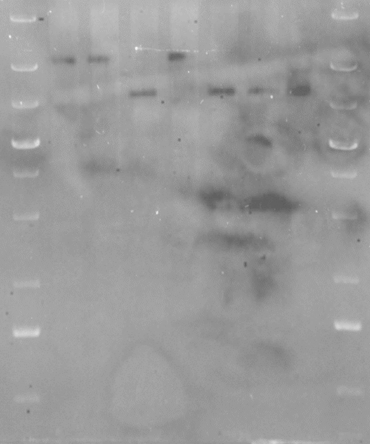


20000 bp

10000 bp

7000 bp

5000 bp

M

MJK20.2

#15

BS6.4

#12

BS6.3

BS6.2

BS6.1

MJK20.2

#15

BS6.4

#12

BS6.3

BS6.2

BS6.1

M

M

M

**A**

**B**

**S1 Figure 4: Deletion of clr2 in MJK20.2.** Shown are the results of the Southern analysis after marker recycling using a 5´probe (A) and a 3´probe (B). Enzymatic digestion was performed using XbaI. A marker (M) was used as a reference for fragment sizes. The expected fragment sizes are shown in S1 Table 2.


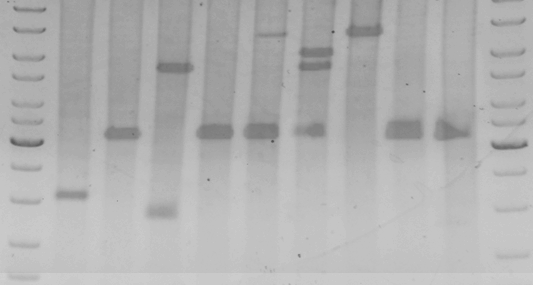

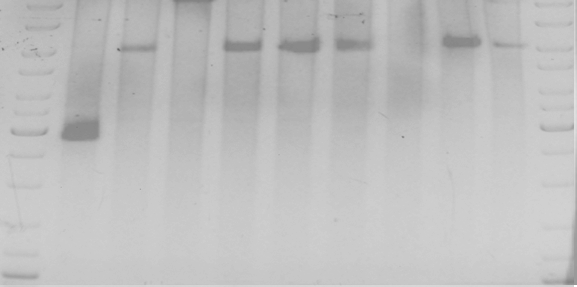


3000 bp

3500 bp

2500 bp

2000 bp

6000 bp

8000 bp

M

M

M

M

MJK20.3

JK2.10

JK2.10

MJK20.3

#9

#9

JK2.8

JK2.8

#6

#6

#5

#5

#3

#3

JK2.2

JK2.2

JK2.1

JK2.1

**A**

**B**

**S1 Figure 5: Deletion of clr4 in MJK20.3.** Shown are the results of the Southern analysis after marker recycling using a 5´probe. Enzymatic digestion was performed using SalI (A) and KpnI (B). A marker (M) was used as a reference for fragment sizes. The expected fragment sizes are shown in S1 Table.

**S1 Table 2: Expected fragment sizes depending on the enzyme used for digestion and the possible genotype.** The parental strain (Δku80) was used as a negative control.

| deleted gene | enzyme | genotype | fragment size [bp] |
| --- | --- | --- | --- |
| *clr2* | XbaI | Δ*ku80* | 11879 |
|  |  | Δ*ku80*, Δ*clr2* | 8180 |
| *clr1* | XmnI | Δ*ku80* | 5660 |
|  |  | Δ*ku80*, Δ*clr1* | 2940 |
| *clr4* | SalI | Δ*ku80* | 2150 |
|  |  | Δ*ku80*, Δ*clr4* | 3274 |
|  | KpnI | Δ*ku80* | 2919 |
|  |  | Δ*ku80*, Δ*clr4* | 6054 |
