# Supplementary material for: Transcriptomic insights into the roles of the transcription factors Clr1, Clr2 and Clr4 in lignocellulose degradation of the thermophilic fungal platform Thermothelomyces thermophilus
Source: Front Bioeng Biotechnol. 2023 Oct 6;11:1279146. doi: 10.3389/fbioe.2023.1279146 (PMC10588483; doi:10.3389/fbioe.2023.1279146)
Supplement: Supplementary file 2 [file Table12.DOCX]

| name | description | reference |
| --- | --- | --- |
| MT121 | puc Ori, 5´*clr2*, P An *gpdA*, An *amdS* part 1, kanR | BASF SE (Ludwigshafen, Germany) |
| MT122 | puc Ori, 5´*clr1*, P An *gpdA*, An *amdS* part 1, kanR | BASF SE (Ludwigshafen, Germany) |
| MT227 | puc Ori, An *amdS* part 2, T An *amdS*, 5´*clr1*, 3´*clr1*, kanR | BASF SE (Ludwigshafen, Germany) |
| MT28 | puc Ori, An *amdS*, ampR; positive control for transformation | BASF SE (Ludwigshafen, Germany) |
| MT497 | puc Ori, An *amdS* part 2, T An *amdS*, 5´*clr2*, 3´*clr2*, kanR | BASF SE (Ludwigshafen, Germany) |
| pBS1.13 | puc Ori, 5´*clr4*, P An *gpdA*, An *amdS,* T An *amdS*, 5´*clr4*, 3´*clr4*, kanR | this study |
| pMJK19.7 | ColE1 Ori, 5´*ku70*, P An *gpdA*, An *amdS*, 3´*ku70*, ampR | Min Jin Kwon, data not published |

**S12 Table 1*:* Plasmids used in this study.** An= *Aspergillus nidulans*.
