# Supplementary material for: Transcriptomic insights into the roles of the transcription factors Clr1, Clr2 and Clr4 in lignocellulose degradation of the thermophilic fungal platform Thermothelomyces thermophilus
Source: Front Bioeng Biotechnol. 2023 Oct 6;11:1279146. doi: 10.3389/fbioe.2023.1279146 (PMC10588483; doi:10.3389/fbioe.2023.1279146)
Supplement: Supplementary file 3 [file Table9.DOCX]

|  |  |  | MJK20.3 | | BS6.4 | | BS7.8 | | JK2.8 | |
| --- | --- | --- | --- | --- | --- | --- | --- | --- | --- | --- |
| **class** | type | genes | t1-t4 up | t1-t4 down | t1-t4 up | t1-t4 down | t1-t4 up | t1-t4 down | t1-t4 up | t1-t4 down |
| **cellulases** | endoglucanases | 9 | 7 | 0 | 2 | 0 | 1 | 0 | 8 | 0 |
|  | cellobiohydrolases | 7 | 5 | 0 | 3 | 0 | 0 | 0 | 5 | 0 |
|  | ß-glucosidases | 9 | 4 | 1 | 4 | 1 | 0 | 1 | 4 | 1 |
|  | LPMOs | 24 | 14 | 0 | 1 | 0 | 0 | 0 | 13 | 0 |
| **hemicellulases** | xylanases | 12 | 5 | 0 | 2 | 0 | 1 | 0 | 5 | 0 |
|  | xylosidases | 4 | 3 | 0 | 2 | 0 | 1 | 0 | 2 | 0 |
|  | endoarabinases | 3 | 0 | 0 | 0 | 0 | 0 | 0 | 0 | 0 |
|  | exoarabinases/ arabinofuranosidases | 11 | 3 | 0 | 2 | 0 | 2 | 0 | 3 | 0 |
|  | mixed-linked glucanase | 5 | 1 | 0 | 0 | 0 | 0 | 1 | 3 | 0 |
|  | mannanases | 10 | 3 | 0 | 1 | 1 | 2 | 1 | 3 | 1 |
|  | mannosidases | 11 | 3 | 1 | 0 | 1 | 0 | 2 | 3 | 2 |
|  | galactanases | 2 | 1 | 0 | 0 | 0 | 0 | 0 | 1 | 0 |
|  | galactosidases | 7 | 3 | 0 | 4 | 0 | 2 | 0 | 3 | 0 |
| **pectinases** | polygalacturonases | 2 | 1 | 0 | 0 | 0 | 0 | 0 | 1 | 0 |
|  | rhamnosidases | 1 | 0 | 0 | 1 | 0 | 1 | 0 | 0 | 0 |
|  | pectin lyases | 8 | 7 | 0 | 2 | 0 | 0 | 0 | 6 | 0 |
| **esterases** | feruloyl esterases | 4 | 0 | 0 | 0 | 0 | 0 | 0 | 0 | 0 |
|  | acetyl esterases | 9 | 5 | 0 | 1 | 0 | 1 | 0 | 5 | 1 |
|  | pectin esterases | 4 | 0 | 0 | 0 | 0 | 0 | 0 | 0 | 0 |
|  | glycuronoyl esterases | 2 | 0 | 0 | 0 | 0 | 0 | 0 | 0 | 0 |
| **starch metabolism** | alpha amylases | 4 | 0 | 2 | 1 | 1 | 2 | 2 | 0 | 1 |
|  | alpha glucosidases | 4 | 0 | 0 | 3 | 0 | 3 | 0 | 0 | 0 |
|  | glucoamylases | 2 | 0 | 1 | 1 | 1 | 1 | 1 | 0 | 1 |
|  | glycogen debranching enzymes | 2 | 0 | 1 | 0 | 1 | 0 | 1 | 0 | 1 |
| **cell wall remodeling** | glucanases | 12 | 1 | 1 | 1 | 3 | 1 | 2 | 0 | 2 |
|  | transglucosylases | 4 | 0 | 1 | 0 | 1 | 0 | 1 | 0 | 2 |
|  | chitosanases | 2 | 1 | 0 | 0 | 0 | 0 | 0 | 1 | 0 |
|  | diacetylmuramidase | 1 | 0 | 0 | 0 | 0 | 0 | 0 | 0 | 1 |
|  | glucosaminidase | 3 | 0 | 0 | 0 | 0 | 0 | 1 | 0 | 0 |
|  | chitinases | 8 | 1 | 0 | 2 | 2 | 3 | 2 | 1 | 0 |
|  | crosslinking transglycosidase | 3 | 0 | 1 | 0 | 1 | 0 | 1 | 0 | 1 |
| **glycosyltransferases** | glycosyltransferases | 83 | 4 | 2 | 8 | 11 | 10 | 17 | 5 | 4 |
| **other** | other | 124 | 24 | 6 | 13 | 6 | 8 | 6 | 20 | 4 |
|  | total | 396 | 96 | 17 | 9 | 8 | 4 | 7 | 12 | 4 |

**S9 Table 1*:* Differentially expressed CAZY categories.** Shown are the numbers of genes belonging to different CAZY classes that are differentially expressed at all points in time after the cellulose spike in strains BS6.4, BS7.8, JK2.8, and the reference strain MJK20.3.
