# Supplementary material for: Transcriptomic insights into the roles of the transcription factors Clr1, Clr2 and Clr4 in lignocellulose degradation of the thermophilic fungal platform Thermothelomyces thermophilus
Source: Front Bioeng Biotechnol. 2023 Oct 6;11:1279146. doi: 10.3389/fbioe.2023.1279146 (PMC10588483; doi:10.3389/fbioe.2023.1279146)
Supplement: Supplementary file 4 [file Table2.DOCX]

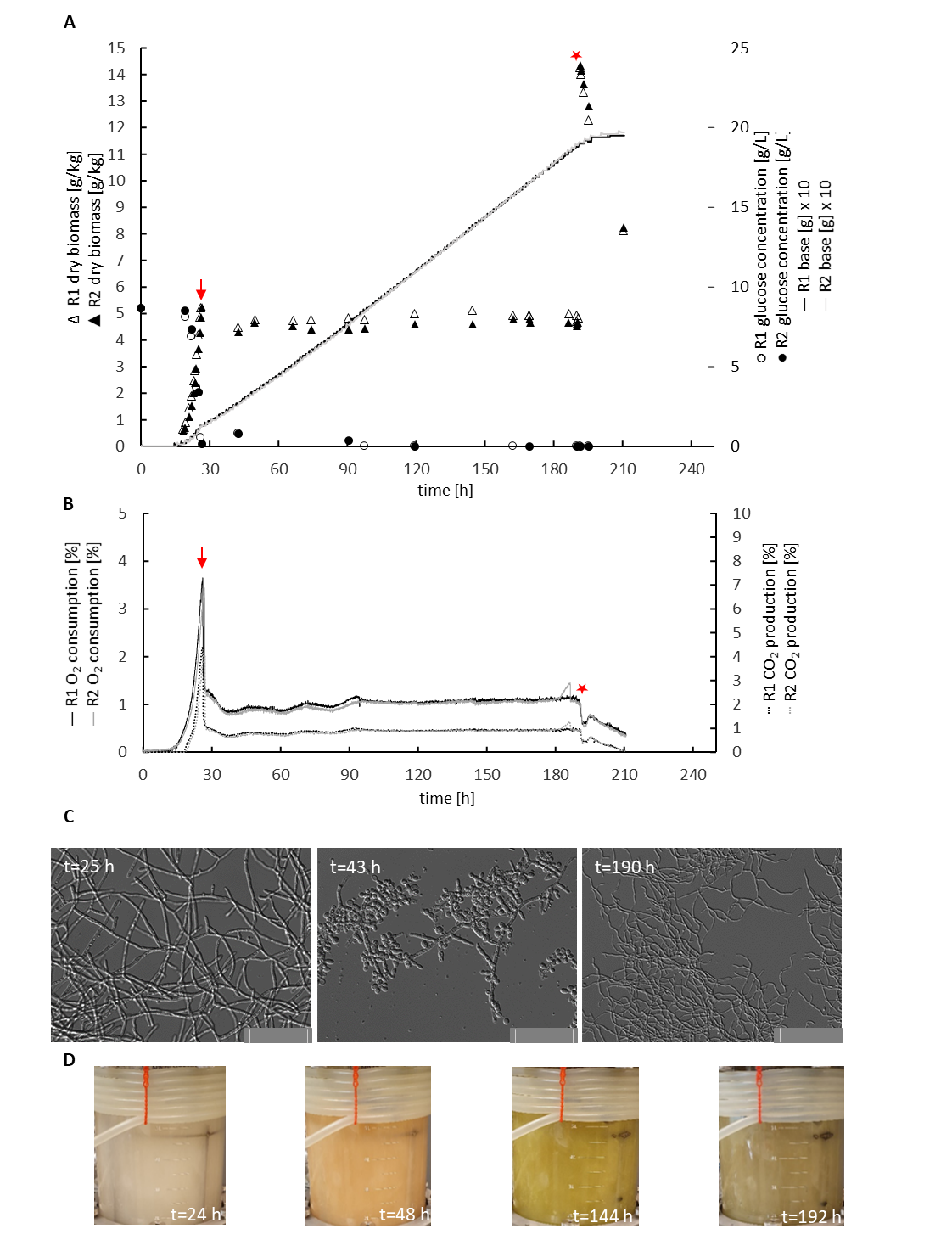


**S2 Figure 1: Physiology of the clr4 deletion strain during chemostat bioreactor cultivation.** Strain JK2.8 was cultivated and analysed as described in Figure legend 2. Biomass accumulation, glucose concentration, base addition (A), oxygen consumption, carbon dioxide production (B), mycelial morphology (C), and colour of the culture broth (D) are given for duplicate cultures of strain JK2.8 (R1, R2). Scale bar= 50 µm.


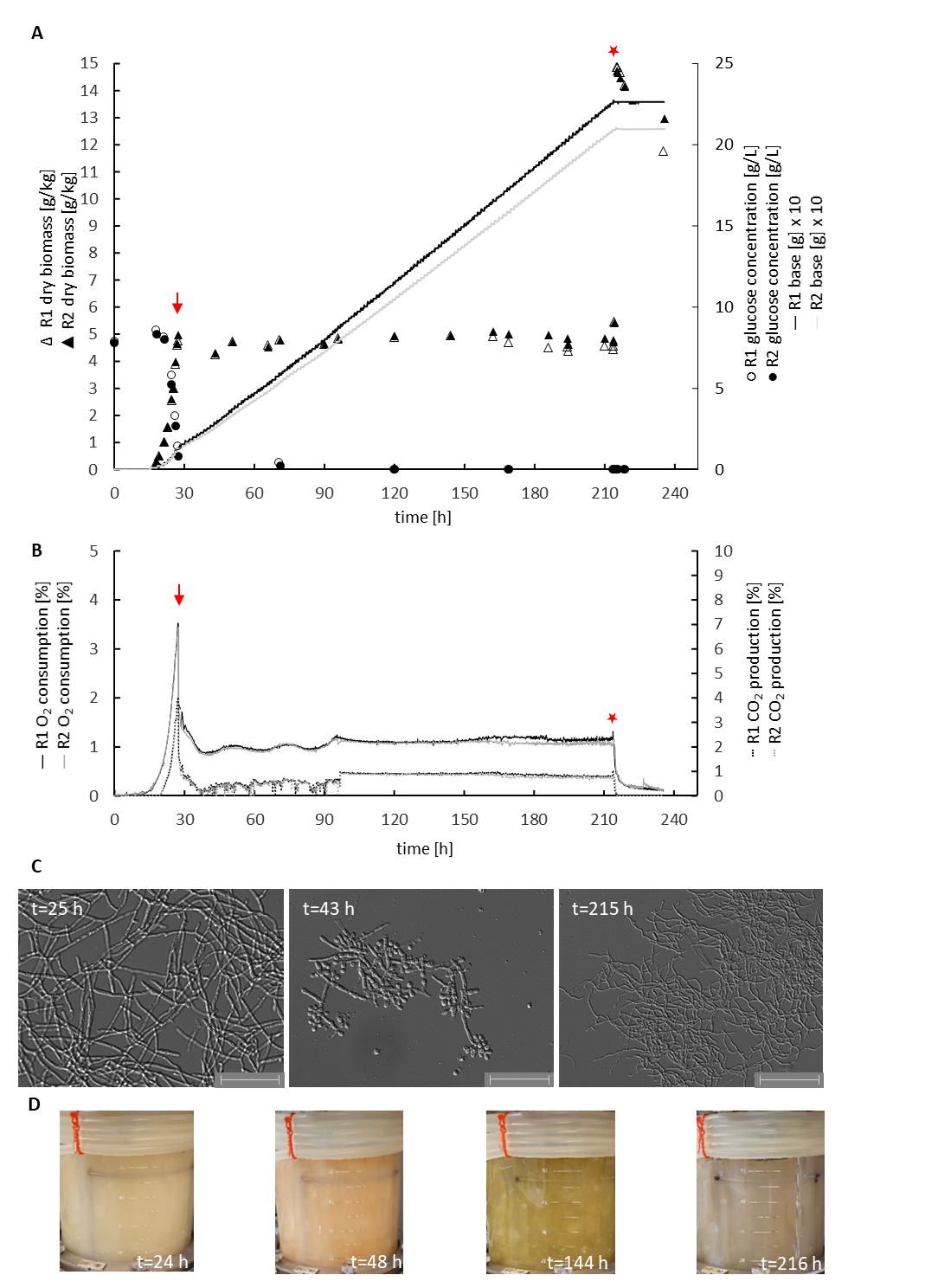


**S2 Figure 2: Physiology of the clr1 deletion strain during chemostat bioreactor cultivation.** Strain BS7.8 was cultivated and analysed as described in Figure legend 2. Biomass accumulation, glucose concentration, base addition (A), oxygen consumption, carbon dioxide production (B), mycelial morphology (C), and colour of the culture broth (D) are given for duplicate cultures of strain BS7.8 (R1, R2). Scale bar= 50 µm.
