# Supplementary material for: Transcriptomic insights into the roles of the transcription factors Clr1, Clr2 and Clr4 in lignocellulose degradation of the thermophilic fungal platform Thermothelomyces thermophilus
Source: Front Bioeng Biotechnol. 2023 Oct 6;11:1279146. doi: 10.3389/fbioe.2023.1279146 (PMC10588483; doi:10.3389/fbioe.2023.1279146)
Supplement: Supplementary file 5 [file Table3.DOCX]

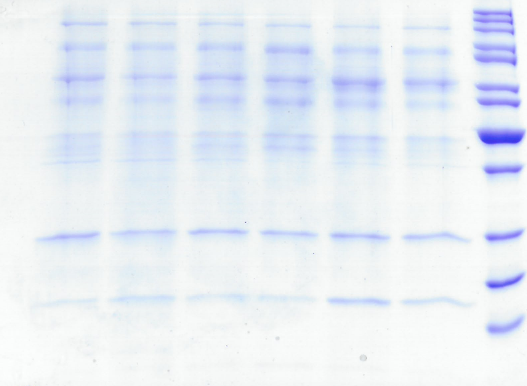

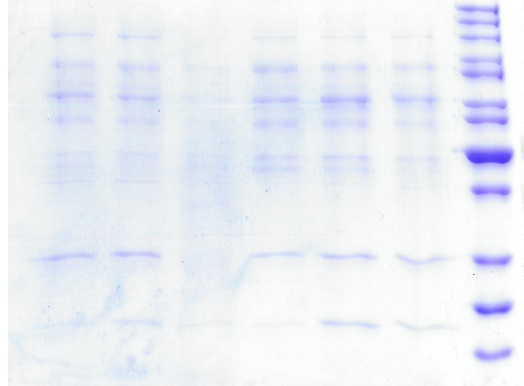

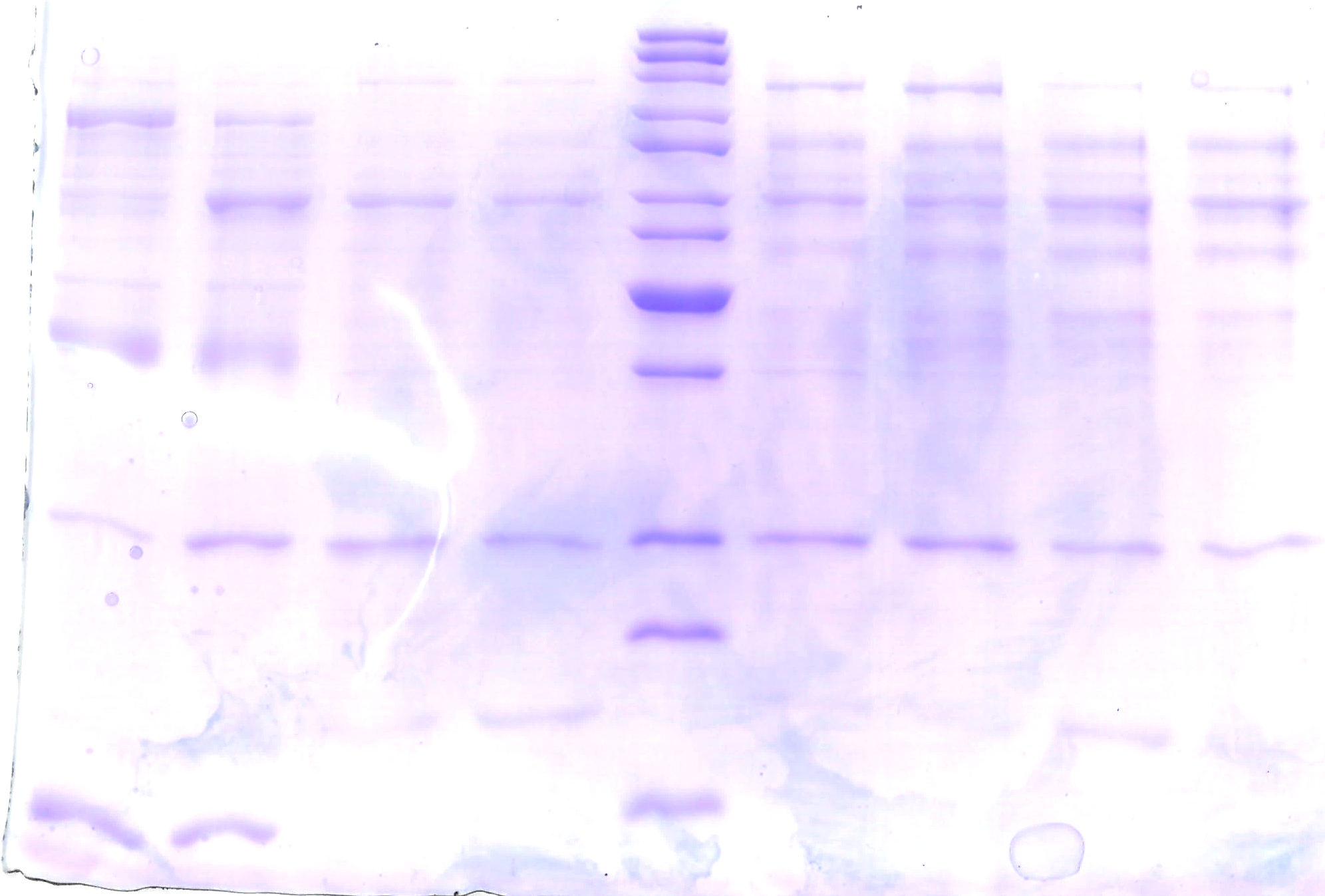

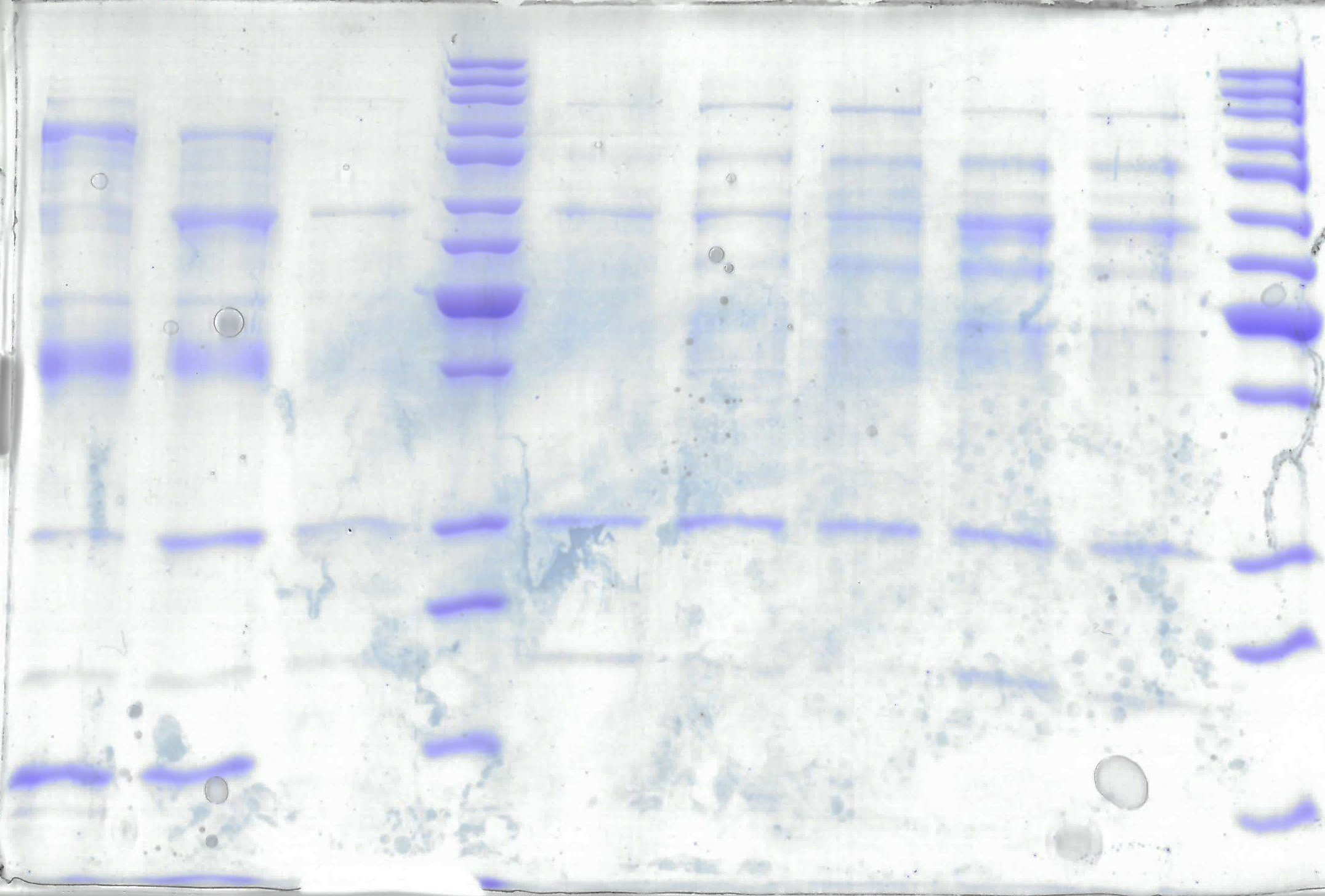


MJK20.3 R1

MJK20.3 R2

BS6.4 R1

BS6.4 R2

MJK20.3 R1

MJK20.3 R2

BS6.4 R1

BS6.4 R2

M

M

M

M

M

MJK20.3 R1

MJK20.3 R2

BS7.8 R1

BS7.8 R2

JK2.8 R1

JK2.8 R2

MJK20.3 R1

MJK20.3 R2

BS7.8 R1

BS7.8 R2

JK2.8 R1

JK2.8 R2

120 kDa

85 kDa

70 kDa

60 kDa

50 kDa

40 kDa

30 kDa

25 kDa

20 kDa

A

B


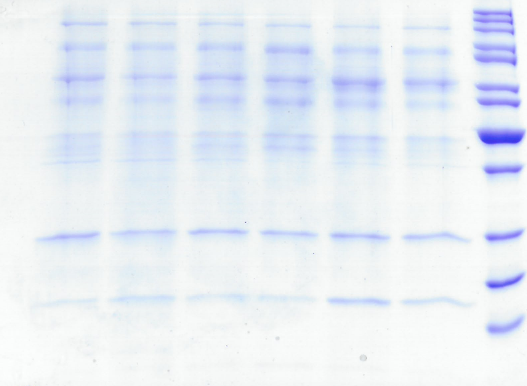

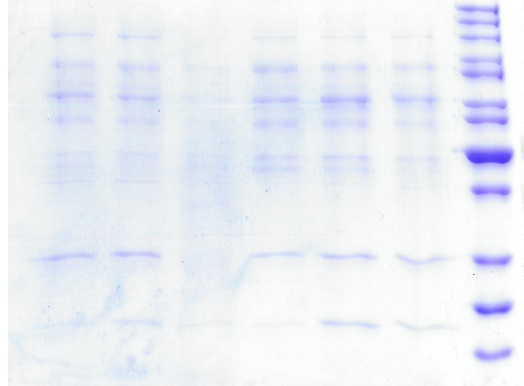


**S3 Figure 1: Protein analysis of culture supernatants at steady state via SDS-PAGE.** Shown are the results of the SDS PAGEs with (A) 2 µg protein (determined via Bradford assay) and (B) 20 µL supernatant for the two replicates (R1, R2) of the strains MJK20.3, BS6.4, BS7.8 and JK2.8 at steady state (SS). As a marker for protein size determination [kDa], PageRuler Unstained Protein Ladder (M) was used.


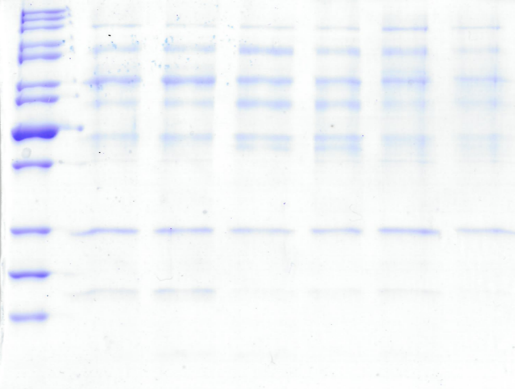

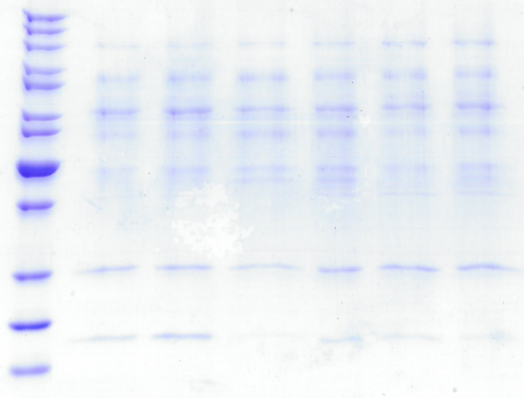


M

M

MJK20.3 R1

MJK20.3 R2

BS6.4 R1

BS6.4 R2

MJK20.3 R1

MJK20.3 R2

BS6.4 R1

BS6.4 R2


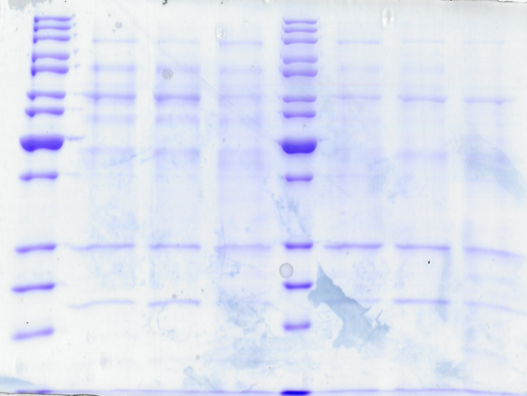

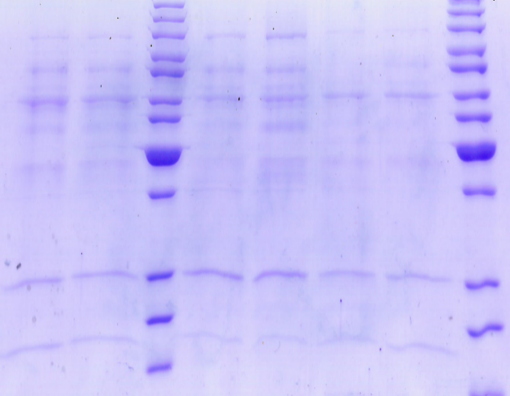


MJK20.3 R1

MJK20.3 R2

BS7.8 R1

BS7.8 R2

JK2.8 R1

JK2.8 R2

MJK20.3 R1

MJK20.3 R2

BS7.8 R1

BS7.8 R2

JK2.8 R1

JK2.8 R2

M

M

M

M

A

B


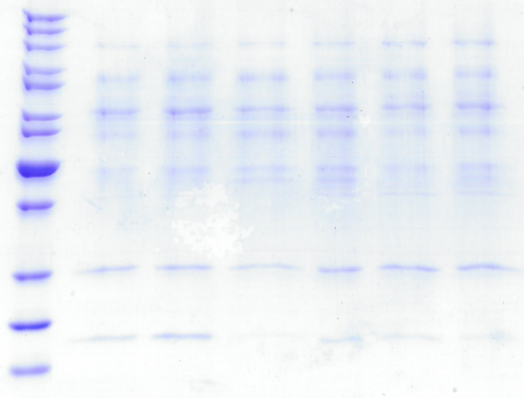

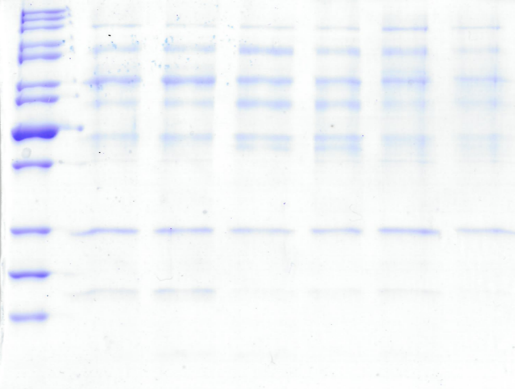


**S3 Figure 2: Protein analysis of culture supernatants 0.5 h after spiking cellulose via SDS-PAGE.** Shown are the results of the SDS PAGEs with (A) 2 µg protein (determined via Bradford assay) and (B) 20 µL supernatant for the two replicates (R1, R2) of the strains MJK20.3, BS6.4, BS7.8 and JK2.8 0.5 h after spiking cellulose (t1). As a marker for protein size determination [kDa], PageRuler Unstained Protein Ladder (M) was used.


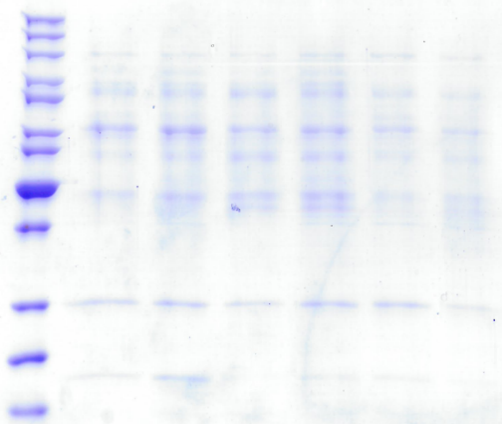

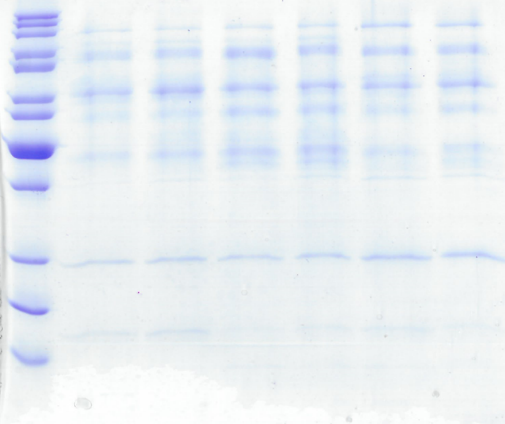


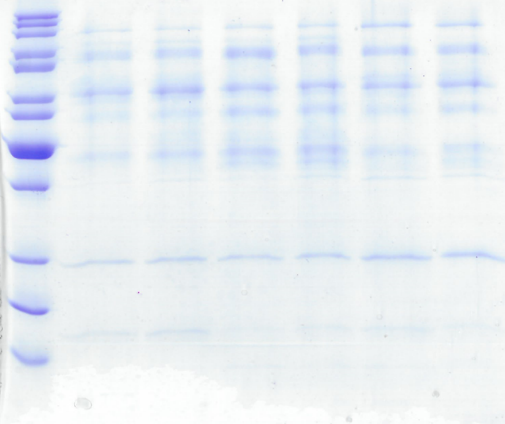

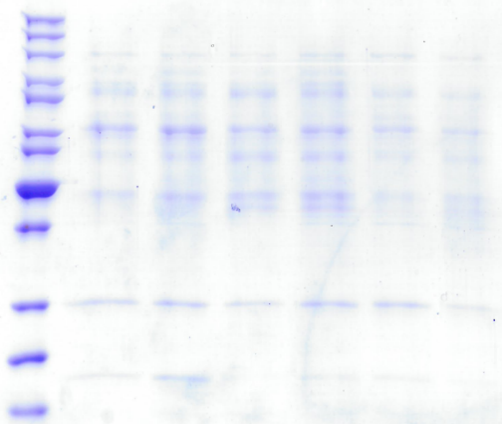

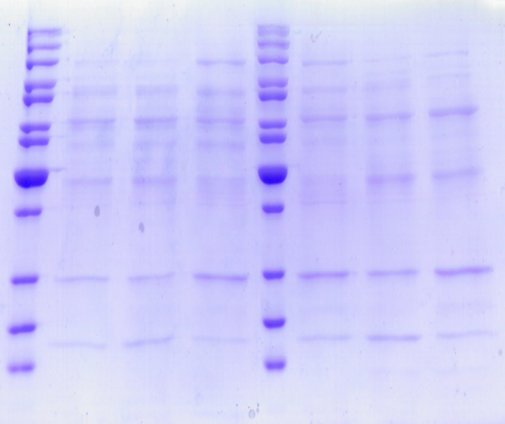

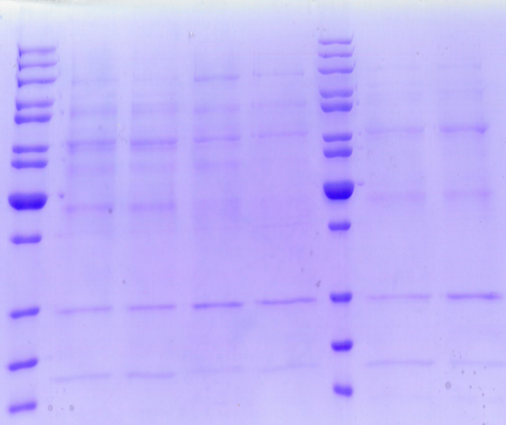


M

M

M

M

M

M

MJK20.3 R1

MJK20.3 R2

BS6.4 R1

BS6.4 R2

MJK20.3 R1

MJK20.3 R2

BS6.4 R1

BS6.4 R2

MJK20.3 R1

MJK20.3 R2

BS7.8 R1

BS7.8 R2

JK2.8 R1

JK2.8 R2

MJK20.3 R1

MJK20.3 R2

BS7.8 R1

BS7.8 R2

JK2.8 R1

JK2.8 R2

A

B

**S3 Figure 3: Protein analysis of culture supernatants 1 h after spiking cellulose via SDS-PAGE.** Shown are the results of the SDS PAGEs with (A) 2 µg protein (determined via Bradford assay) and (B) 20 µL supernatant for the two replicates (R1, R2) of the strains MJK20.3, BS6.4, BS7.8 and JK2.8 1 h after spiking cellulose (t2). As a marker for protein size determination [kDa], PageRuler Unstained Protein Ladder (M) was used.


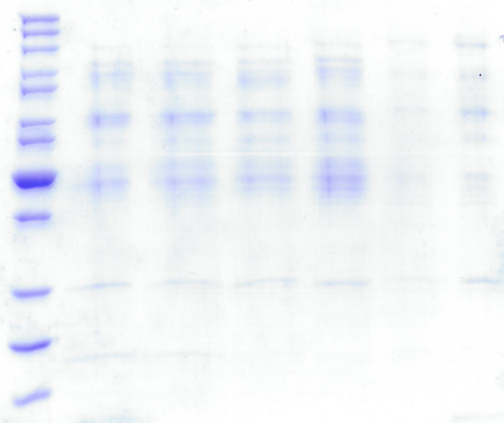

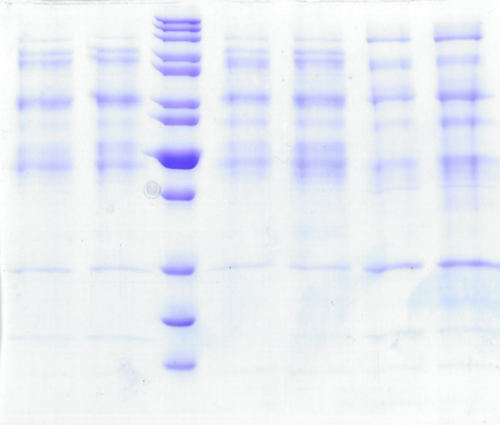


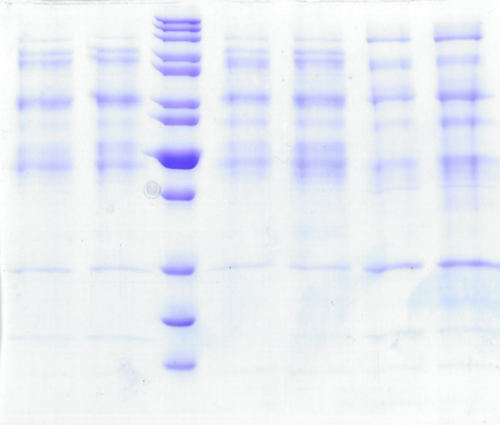

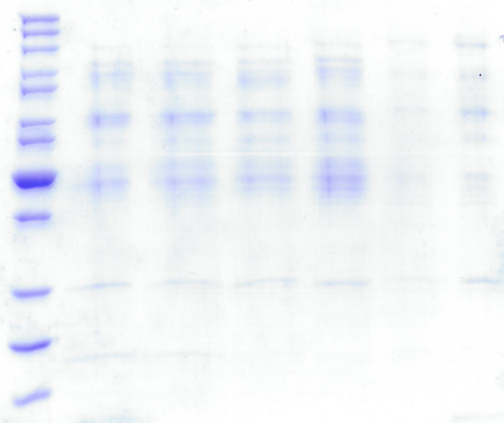

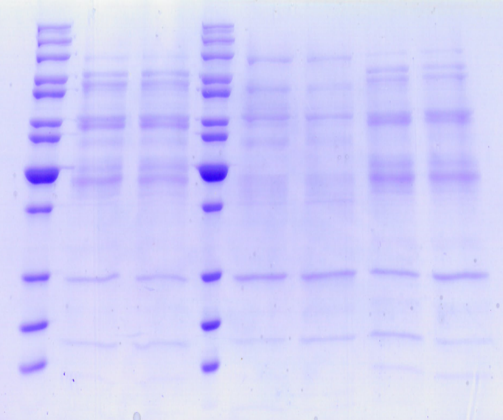

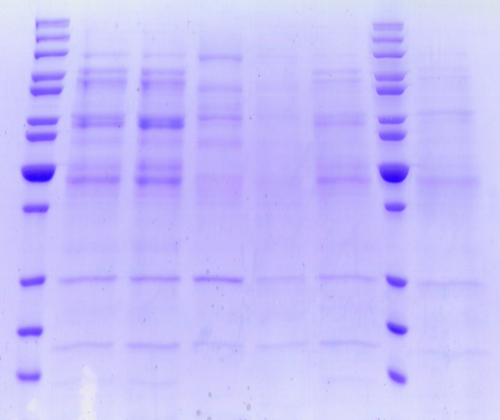


M

M

M

M

M

M

MJK20.3 R1

MJK20.3 R2

BS6.4 R1

BS6.4 R2

MJK20.3 R1

MJK20.3 R2

BS6.4 R1

BS6.4 R2

MJK20.3 R1

MJK20.3 R2

BS7.8 R1

BS7.8 R2

JK2.8 R1

JK2.8 R2

MJK20.3 R1

MJK20.3 R2

BS7.8 R1

BS7.8 R2

JK2.8 R1

JK2.8 R2

A

B

**S3 Figure 4: Protein analysis of culture supernatants 2 h after spiking cellulose via SDS-PAGE.** Shown are the results of the SDS PAGEs with (A) 2 µg protein (determined via Bradford assay) and (B) 20 µL supernatant for the two replicates (R1, R2) of the strains MJK20.3, BS6.4, BS7.8 and JK2.8 2 h after spiking cellulose (t3). As a marker for protein size determination [kDa], PageRuler Unstained Protein Ladder (M) was used.


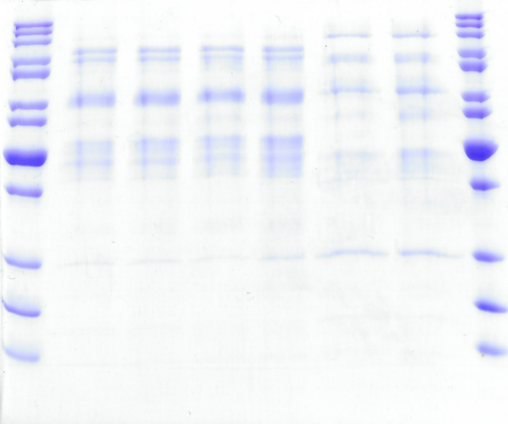

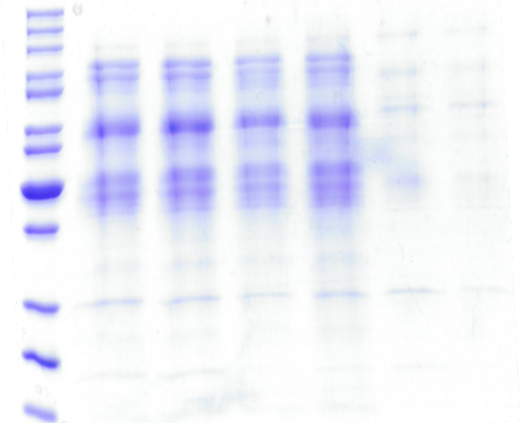

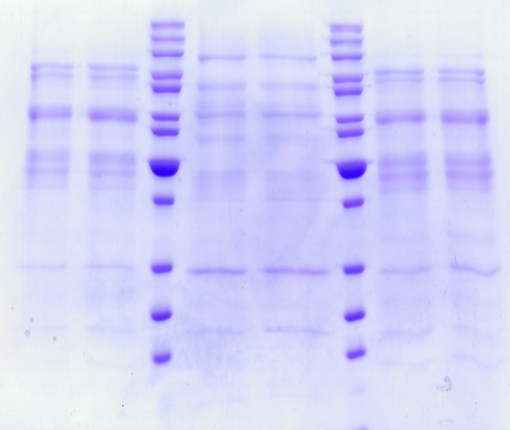

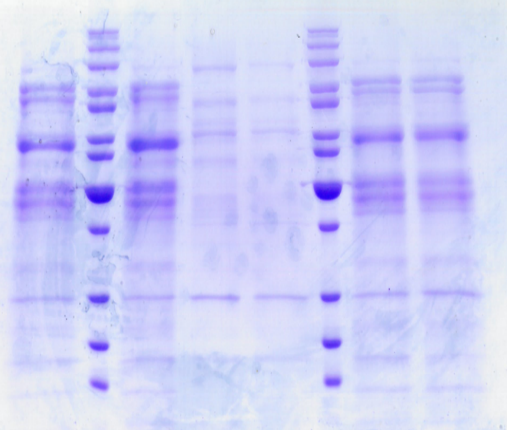


M

M

M

M

M

M

MJK20.3 R1

MJK20.3 R2

BS6.4 R1

BS6.4 R2

MJK20.3 R1

MJK20.3 R2

BS6.4 R1

BS6.4 R2

MJK20.3 R1

MJK20.3 R2

BS7.8 R1

BS7.8 R2

JK2.8 R1

JK2.8 R2

MJK20.3 R1

MJK20.3 R2

BS7.8 R1

BS7.8 R2

JK2.8 R1

JK2.8 R2

A

B


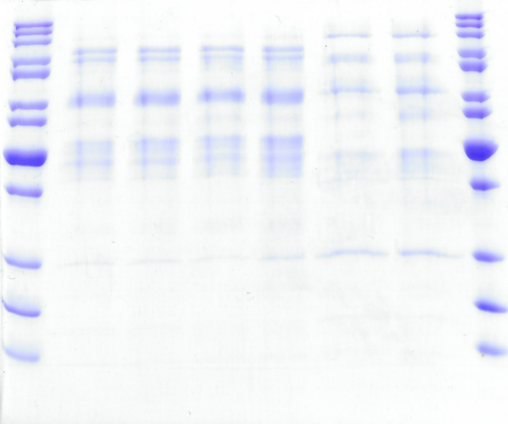

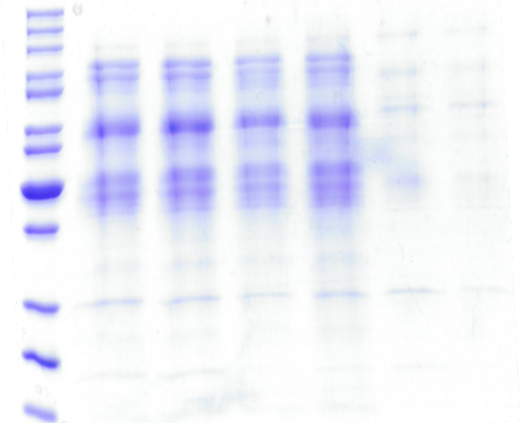


M

**S3 Figure 5: Protein analysis of culture supernatants 4 h after spiking cellulose via SDS-PAGE.** Shown are the results of the SDS PAGEs with (A) 2 µg protein (determined via Bradford assay) and (B) 20 µL supernatant for the two replicates (R1, R2) of the strains MJK20.3, BS6.4, BS7.8 and JK2.8 4 h after spiking cellulose (t4). As a marker for protein size determination [kDa], PageRuler Unstained Protein Ladder (M) was used.
