# Supplementary material for: Transcriptomic insights into the roles of the transcription factors Clr1, Clr2 and Clr4 in lignocellulose degradation of the thermophilic fungal platform Thermothelomyces thermophilus
Source: Front Bioeng Biotechnol. 2023 Oct 6;11:1279146. doi: 10.3389/fbioe.2023.1279146 (PMC10588483; doi:10.3389/fbioe.2023.1279146)
Supplement: Supplementary file 6 [file Table4.DOCX]

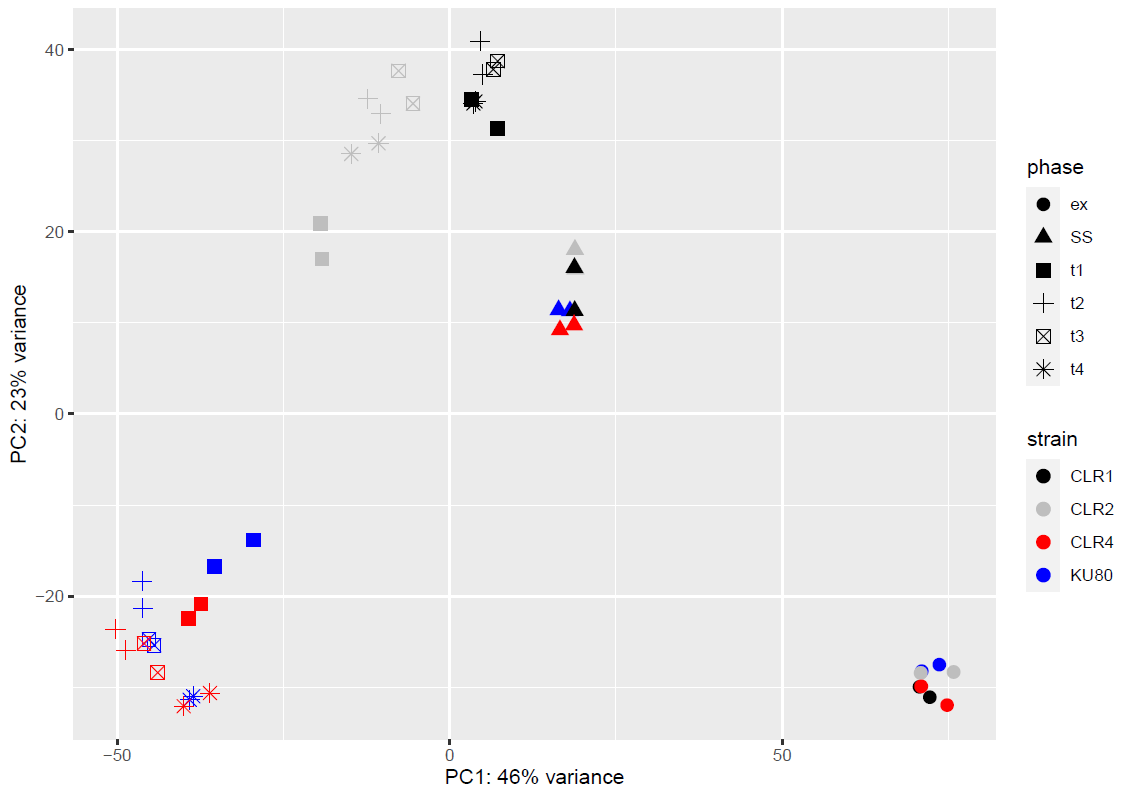


**S4 Figure 1: Principal component analysis (PCA).** Shown are the similarities/relations of the expression profiles of all analyzed samples. The closer the datapoints for each strain or condition are relatively to each other, the more similar is their expression profile. A higher distance in principal component 1 (PC1; 46 % variance) means a higher variance between the samples compared to the same distance in principal component 2 (PC2; 23 % variance). Samples analyzed include the conditions exponential state (ex), steady state (SS), as well as 0.5 h (t1), 1 h (t2), 2 h (t3), and 4 h (t4) after spiking cellulose.


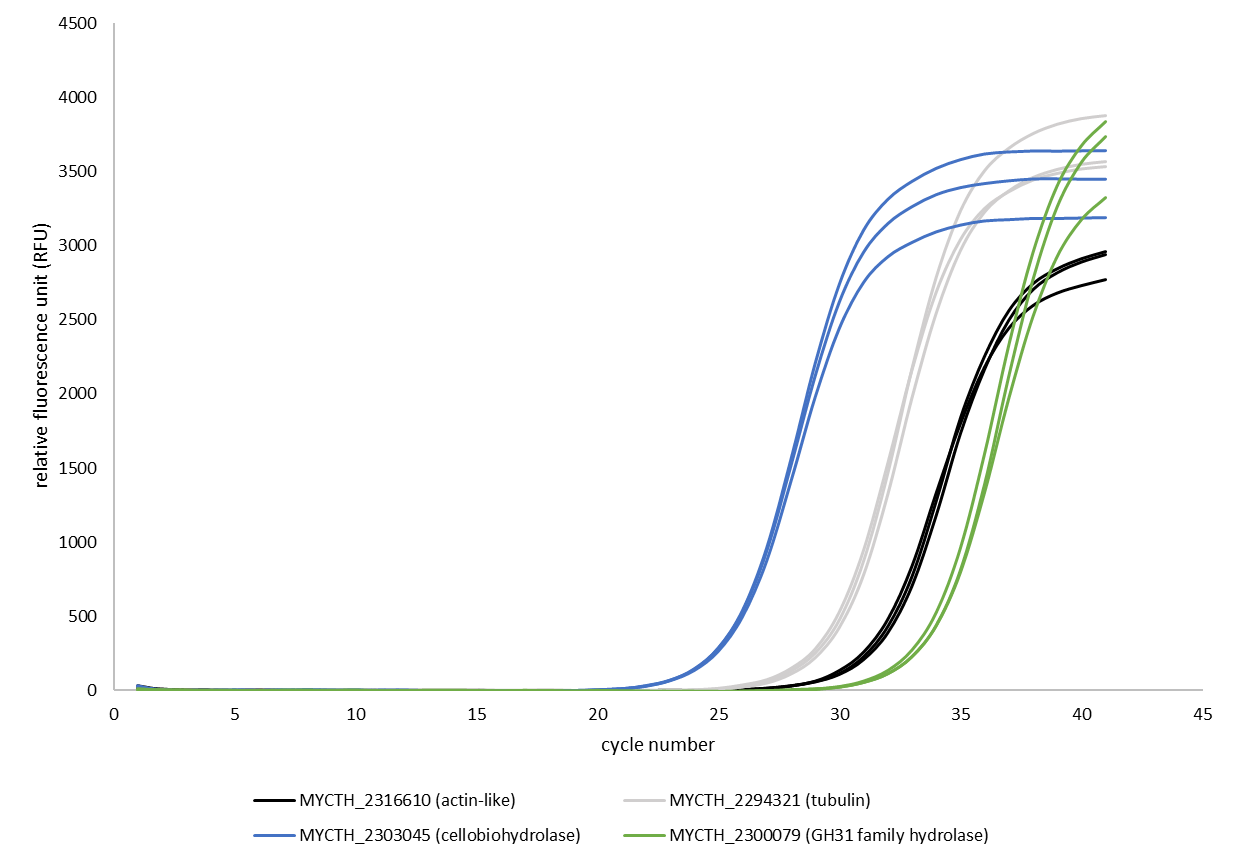


**S4 Figure 2: Quantitative real-time PCR for confirmation of the quality of the RNA seq. analysis.** Shown are the relative fluorescence units (RFU) by cycle number. The genes that were examined include MYCTH_2316610 (actin-like), MYCTH_2294321 (tubulin), MYCTH_2303045 (cellobiohydrolase), and MYCTH_2300079 (GH31 family hydrolase).

**S4 Table 1: RNA seq. normalized raw counts of the qPCR target genes in sample MJK20.3 R1, t2.**

| target gene | counts in sample MJK20.3 R1, t2 |
| --- | --- |
| MYCTH_2316610 (actin-like) | 1107 |
|  |  |
| MYCTH_2294321 (tubulin) | 1829 |
|  |  |
| MYCTH_2303045 (cellobiohydrolase) | 48232 |
|  |  |
| MYCTH_2300079 (GH 31 family hydrolase) | 240 |
|  |  |
