# Supplementary material for: Transcriptomic insights into the roles of the transcription factors Clr1, Clr2 and Clr4 in lignocellulose degradation of the thermophilic fungal platform Thermothelomyces thermophilus
Source: Front Bioeng Biotechnol. 2023 Oct 6;11:1279146. doi: 10.3389/fbioe.2023.1279146 (PMC10588483; doi:10.3389/fbioe.2023.1279146)
Supplement: Supplementary file 7 [file Table11.DOCX]

| number | sequence (5´🡪 3´) | description |
| --- | --- | --- |
| 1619 | AAATCCCGTAGCGGCCG | *amdS* part 1 rev primer, amplification of split marker deletion cassette 1 |
| 1620 | AAATCCCGTAGCGGCCGCA | amplification of split marker deletion cassette 2 from MT227 together with primer 1624 |
| 1624 | AAATCTCGAGAGGCCTGACGTCG | amplification of split marker deletion cassette 2 from MT227 together with primer 1620 |
| 1637 | AATCTCGAGAGGCCTGACG | amplification of split marker deletion cassette 1 from MT122 together with primer 1619 |
| 1657 | CATGCACGCCCGTAATGAAG | amplification of split marker deletion cassette 1 from MT121 together with primer 1619 |
| 1658 | GACTCGGTTCTGACAACCATG | *amdS* part 2 fw primer, amplification of split marker deletion cassette 2 |
| 1659 | GTACTCTCTGCTTCACCATCG | amplification of split marker deletion cassette 2 from MT497 together with primer 1658 |

**S11 Table 1*:* Oligonucleotides for the amplification of split marker deletion cassettes.**

**S11 Table 2*:* Oligonucleotides used for the diagnostic PCR of transformants.**

| number | sequence (5´🡪3´) | description |
| --- | --- | --- |
| 1681 | CCACCAGGGCTACGAAACATC | An *amdS*, fw primer |
| 1817 | CAAGAGATGCCAAATGCAGG | *clr2* 3´Locus, rev primer |
| 1734 | CATGATGACGGGCTGGGTTC | *clr2* ORF, fw primer |
| 1687 | CCACAGCGGAGCATCAGGC | *clr2* 5´Locus, fw primer |
| 1733 | GTTCTTTCTCCCAGCCTCAGC | *clr1* ORF, fw primer |
| 1691 | GATGGAAACACTTCTCCGATC | *clr1* 3´Locus, rev primer |
| 1686 | CCATGTTGCGGACTTGCTCC | *clr1* 5´Locus, fw primer |
| 2163 | GAAGGACTCAAAGCCGAAGC | *clr4* ORF, fw primer |
| 2162 | CGTACAGTGTCAAGGCAAATGG | *clr4* 3´Locus, rev primer |
| 2161 | CAGTAGCCGTGGTAGGACG | *clr4* 5´Locus, rev primer |

**S11 Table 3*:* Oligonucleotides used for the amplification of probes for Southern analysis of transformants.**

| number | sequence (5´🡪3´) | description |
| --- | --- | --- |
| 1741 | CTAAGACCCACACCACCACC | amplification of 5´ flank probe for Southern analysis of potential *clr2* deletion clones |
| 1742 | GTAGTGTAACAGGCGAGAGCG |  |
| 1743 | CTCGGGTTCACCTTTCAAATGC | amplification of 3´ flank probe for Southern analysis of potential *clr2* deletion clones |
| 1744 | GATTTGTGGTTGGCGGCTG |  |
| 1737 | GTTGGATGGAATGCTCGGACC | amplification of 5´ flank probe for Southern analysis of potential *clr1* deletion clones |
| 1738 | GAGCTGCACAACGCGATCAG |  |
| 1739 | CGCAGCGAGTCACAATTTGG | amplification of 3´ flank probe for Southern analysis of potential *clr1* deletion clones |
| 1740 | GTGGCTTGATCTCATGGACGC |  |
| 2081 | GAGCTCGAATTGGACGCTGATGGTATGTCAACGGTTGATCC | amplification of 5´ flank probe for Southern analysis of potential *clr4* deletion clones |
| 2128 | CGTAGGTAAGCAAGATCATCG |  |

**S11 Table 4*:* Oligonucleotides used for the amplification of guide RNA templates.**

| number | sequence (5´🡪3´) | description |
| --- | --- | --- |
| 1697 | ATGTAATACGACTCACTATAGGTAATTTCTACTGTTGTAGAT | general fw primer for amplification of the sgRNA template carrying T7 promotor sequence and direct repeat |
| 2048 | CTTCTTTGATGATTTCAGCGATCTACAACAGTAGAAATTA | amplification of a sgRNA template for the deletion of *clr1* via CRISPR/Cas12a together with primer 1697 |
| 2049 | CTCAAATTCTCCTCCAGCTTATCTACAACAGTAGAAATTA | amplification of a sgRNA template for the deletion of *clr1* via CRISPR/Cas12a together with primer 1697 |
| 2152 | GGTCAGTGGTGAATGCGCGCTGTATCTACAACAGTAGAAATTA | amplification of the sgRNA template for the deletion of *clr4* via CRISPR/Cas12a together with primer 1697 |

| number | sequence (5´🡪3´) | description |
| --- | --- | --- |
| 2128 | CGTAGGTAAGCAAGATCATCG | amplification of 5´flank fragment A for CPEC |
| 2081 | GAGCTCGAATTGGACGCTGATGGTATGTCAACGGTTGATCC |  |
| 2131 | ACTGATTGATGACGGCTGAGG | amplification of 3´flank fragment for CPEC |
| 2087 | GGACTGGCTTTCTACGTGTTCGTGTTTTCCACGAAGCGAGC |  |
| 2130 | CGTAGGTAAGCAAGATCATCG | amplification of 5´flank fragment B for CPEC |
| 2085 | CCTCAGCCGTCATCAATCAGTTGGTATGTCAACGGTTGATCC |  |
| 2129 | TCAGCGTCCAATTCGAGCTC | amplification of *amdS* fragment for CPEC |
| 2083 | CGATGATCTTGCTTACCTACGCATGGGTTGAGTGGTATGG |  |
| 2132 | GAACACGTAGAAAGCCAGTCC | amplification of backbone fragment for CPEC |
| 2089 | CGATGATCTTGCTTACCTACGGACGTCAGGCCTCTCGAGAT |  |
| 2136 | GAGCTCGAATTGGACGCTGA | fusion of backbone and 5´ flank fragment A together with primer 2132 |
| 2139 | GGACTGGCTTTCTACGTGTTC | fusion of 3´flank and 5´ flank fragment B together with primer 2130 |
| 2145 | CTCCATATTCTCCGATGATGC | primers used for sequencing of the resulting plasmid after CPEC |
| 2146 | GGCACAAGTGTCTCTCACC |  |
| 2147 | GAATCCCAATCTTAACGCTACC |  |
| 2148 | CATTCGACGTAACAGCTCG |  |
| 2149 | GCAAGCAGCAGATTACGC |  |

**S11 Table 5*:* Oligonucleotides used for the amplification of fragments for CPEC and analysis of the resulting plasmid.**

**S11 Table 6*:* Oligonucleotides used for qPCR.**

| number | sequence | description |
| --- | --- | --- |
| 2587 | CTCGTCGATCAAATCGATCC | primer for qPCR; target gene: MYCTH_2316610 |
| 2588 | CGACGTGCTTCAGGAAC |  |
| 2589 | GATTGCCCAGGTCGTCTC | primer for qPCR; target gene: MYCTH_2294321 |
| 2590 | GCTCGAAGCACTGGAAG |  |
| 2591 | CAAGACGCAGTTCGTCAAC | primer for qPCR; target gene: MYCTH_2303045 |
| 2592 | CTCCTGCCCGTCTTCTTC |  |
| 2593 | CAAGGCAGCGTACAAGG | primer for qPCR; target gene: MYCTH_2300079 |
| 2594 | GTCGACCTCGTGATGATG |  |
