# Supplementary material for: Transcriptomic insights into the roles of the transcription factors Clr1, Clr2 and Clr4 in lignocellulose degradation of the thermophilic fungal platform Thermothelomyces thermophilus
Source: Front Bioeng Biotechnol. 2023 Oct 6;11:1279146. doi: 10.3389/fbioe.2023.1279146 (PMC10588483; doi:10.3389/fbioe.2023.1279146)
Supplement: Supplementary file 8 [file Table6.DOCX]

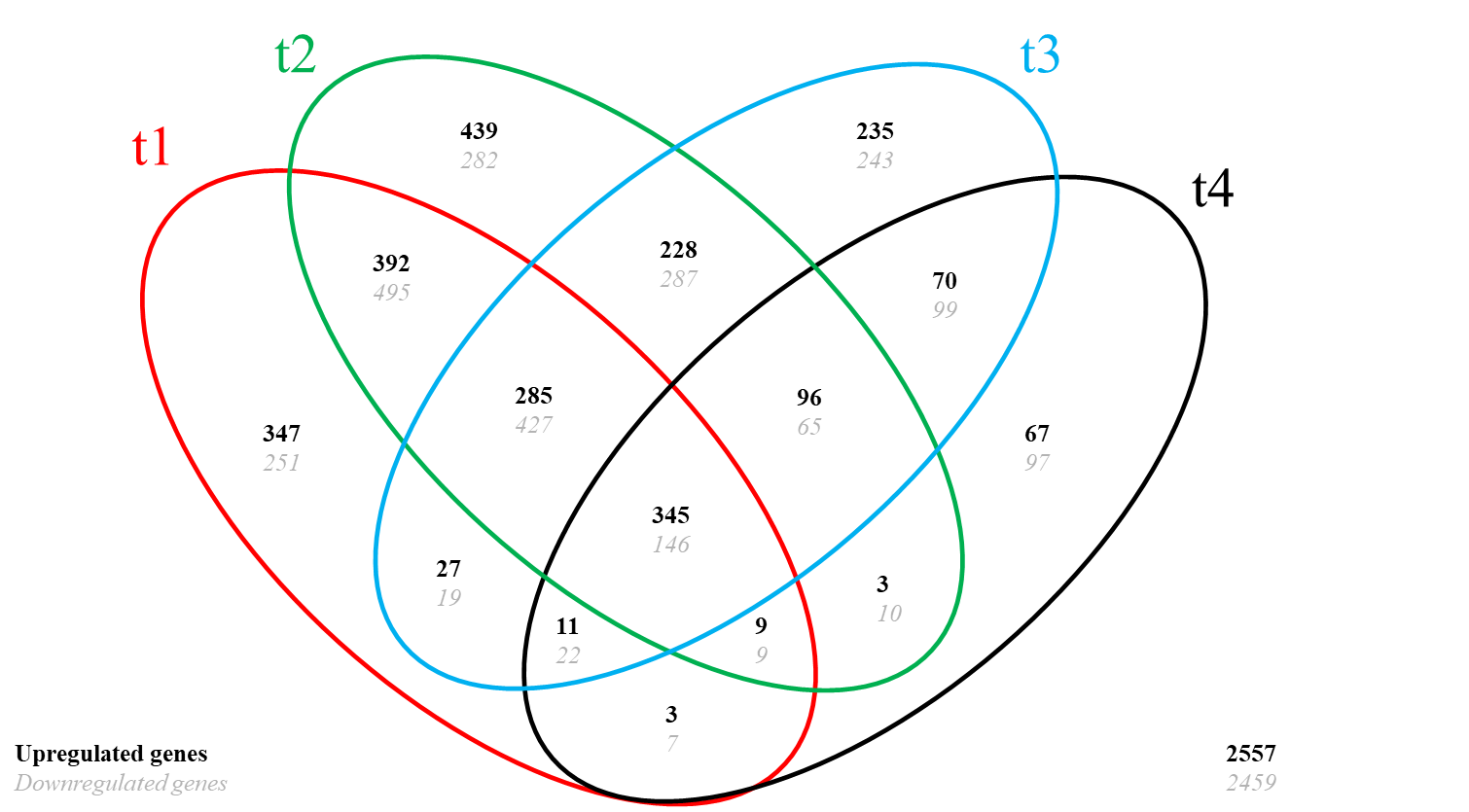


MJK20.3

A


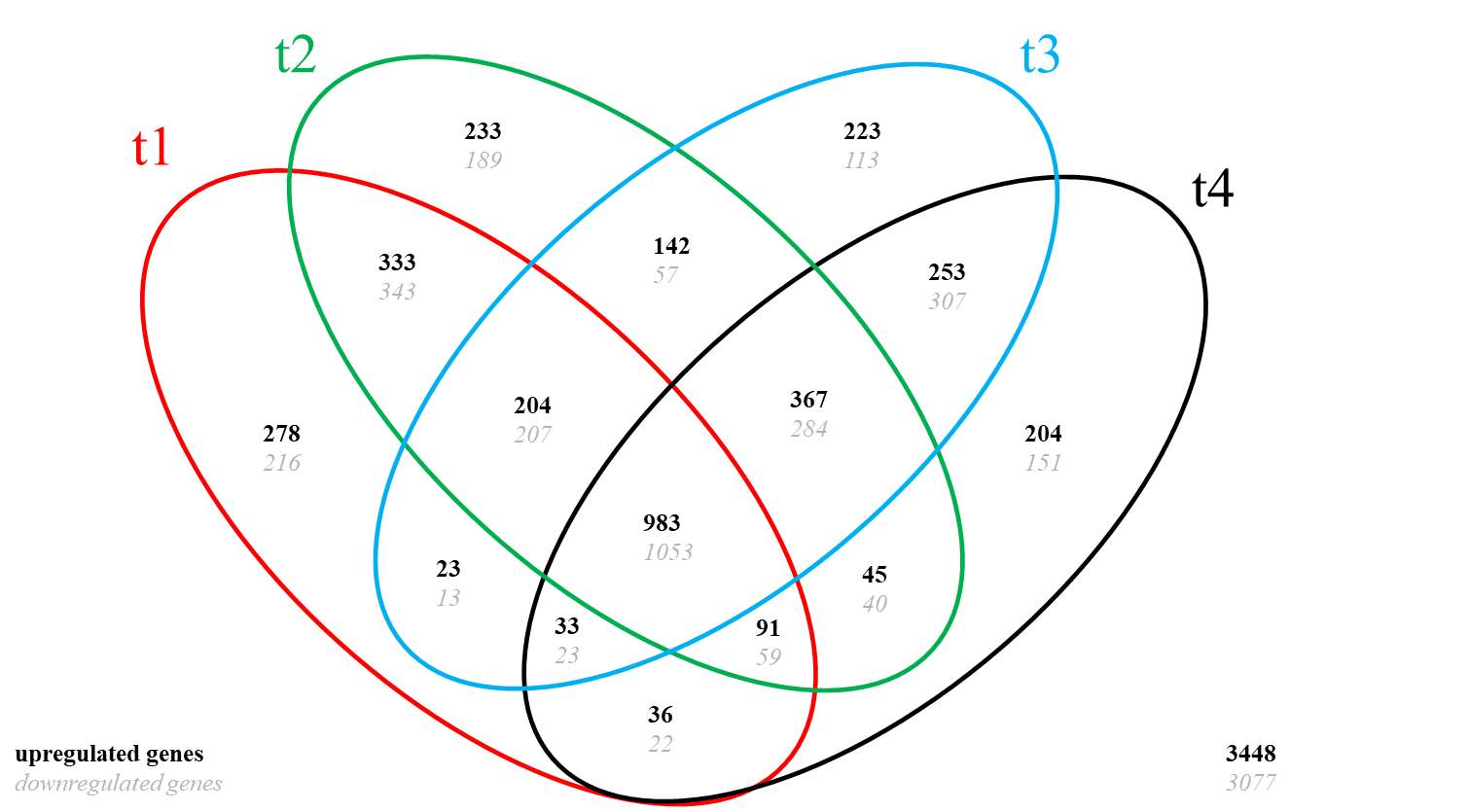


BS6.4

B

BS7.8


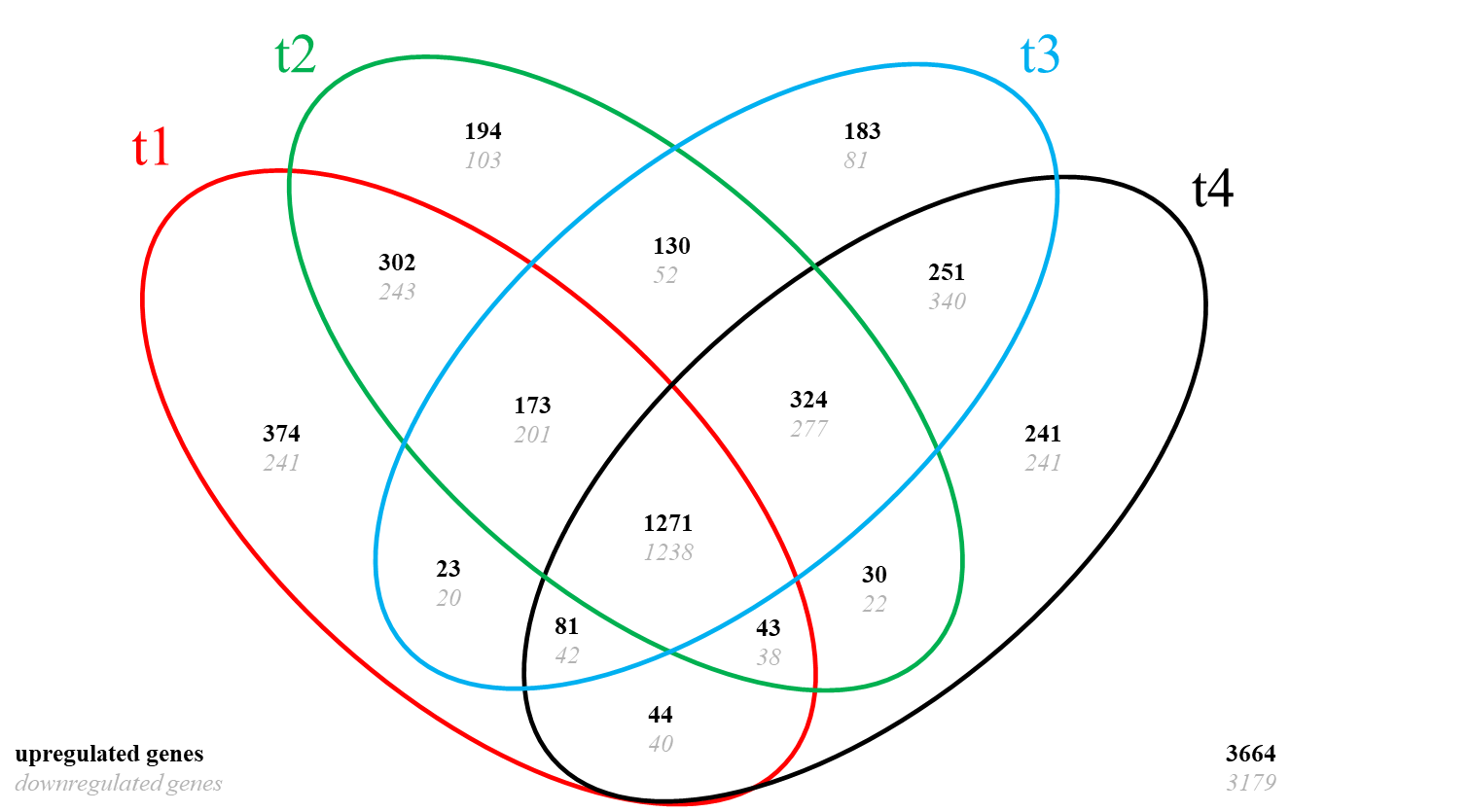


C

JK2.8


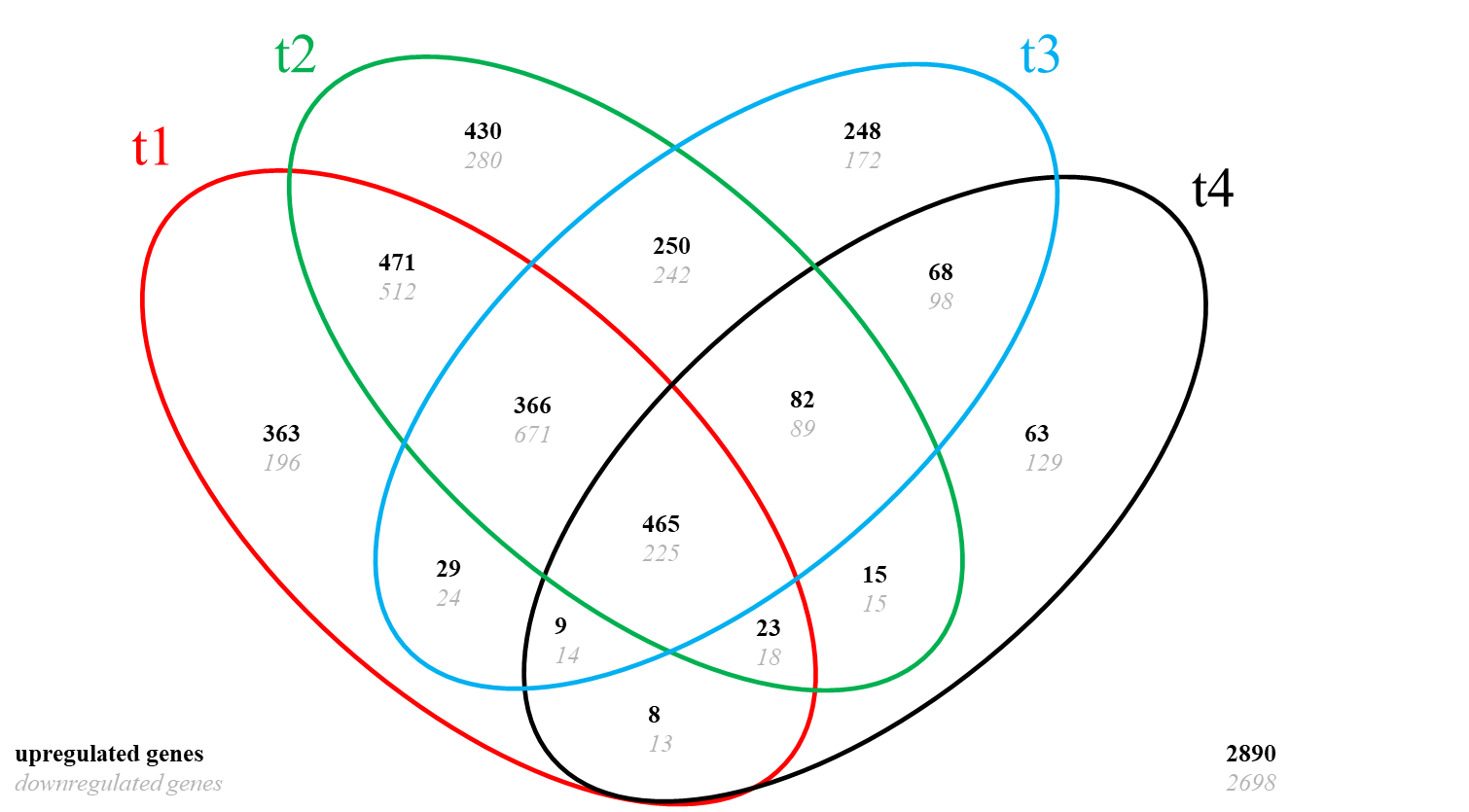


D

**S6 Figure 1*:* Venn diagrams of differentially expressed genes after spiking cellulose.** Black numbers refer to up- and grey numbers to downregulated genes in strains MJK20.3 (A), BS6.4 (B), BS7.8 (C), and JK2.8 (D). The total number of uniquely up- and downregulated genes is shown in the bottom right corner of (A), (B), (C), and (D). Differential expression (padj. ≤ 0 05) was determined via comparison of expression profiles of t1= 0.5 h, t2= 1 h, t3= 2 h, and t4= 4 h after spiking cellulose with the respective steady state condition.
